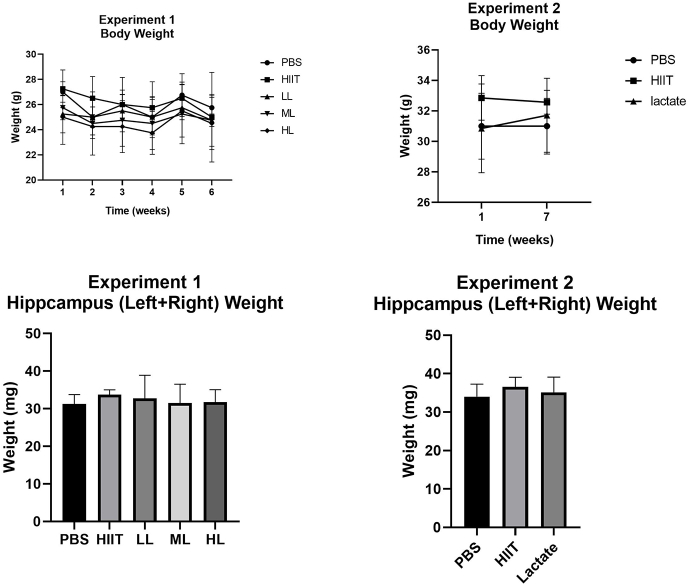# The effects of long-term lactate and high-intensity interval training (HIIT) on brain neuroplasticity of aged mice

**DOI:** 10.1016/j.heliyon.2024.e24421

**Published:** 2024-01-10

**Authors:** Zhou Lei, Soroosh Mozaffaritabar, Takuji Kawamura, Atsuko Koike, Attila Kolonics, Johanna Kéringer, Ricardo A. Pinho, Jingquan Sun, Ruonan Shangguan, Zsolt Radák

**Affiliations:** a Research Institute of Molecular Exercise Science, Hungarian University of Sports Science, H-1123, Budapest, Hungary; b Waseda Institute for Sport Sciences, Waseda University, Saitama, 359-1192, Japan; c Department of Life Sciences, Graduate School of Arts and Sciences, The University of Tokyo, Tokyo, 153–8902, Japan; d Laboratory of Exercise Biochemistry in Health, Graduate Program in Health Sciences, School of Medicine, Pontifícia Universidade Católica do Paraná, Curitiba, 80215-901, Brazil; e Institute of Sports Science, Sichuan University, No. 17, Section 3, Renmin South Road, Chengdu, China; f Department of Physical Education, Chengdu University, 610106, Chengdu, China

**Keywords:** Lactate, Exercise, Aging, Brain function, Lactylation, Hippocampus

## Abstract

Extensive research has confirmed numerous advantages of exercise for promoting brain health. More recent studies have proposed the potential benefits of lactate, the by-product of exercise, in various aspects of brain function and disorders. However, there remains a gap in understanding the effects of lactate dosage and its impact on aged rodents. The present study first examined the long-term effects of three different doses of lactate intervention (2000 mg/kg, 1000 mg/kg, and 500 mg/kg) and high-intensity interval training (HIIT) on aging mice (20–22 months) as the 1st experiment. Subsequently, in the 2nd experiment, we investigated the long-term effects of 500 mg/kg lactate intervention and HIIT on brain neuroplasticity in aged mice (25–27 months).

The results of the 1st experiment demonstrated that both HIIT and different doses of lactate intervention (500 mg/kg and 2000 mg/kg) positively impacted the neuroplasticity biomarker VEGF in the hippocampus of aging mice. Subsequently, the 2nd experiment revealed that long-term HIIT significantly improved the performance of mice in open-field, novel object recognition, and passive avoidance tests. However, lactate intervention did not significantly affect these behavioral tests. Moreover, compared to the control group, both HIIT and lactate intervention positively influenced the angiogenesis signaling pathway (p/t-AKT/ENOS/VEGF), mitochondrial biomarker (SDHA), and metabolic protein (p/t-CREB, p/t-HSL, and LDH) in the hippocampus of aged mice. Notably, only lactate intervention significantly elevated the BDNF (PGC-1α, SIRT1, and BDNF) signaling pathway and metabolic content (lactate and pyruvate). In the end, long-term HIIT and lactate intervention failed to change the protein expression of p/t-MTOR, iNOS, nNOS, HIF-1α, SYNAPSIN, SIRT3, NAMPT, CS, FNDC5 and Pan Lactic aid-Lysine in the hippocampus of aged mice.

In summary, the present study proved that long-term HIIT and lactate treatment have positive effects on the brain functions of aged mice, suggesting the potential usage of lactate as a therapeutic strategy in neurodegenerative diseases in the elderly population.

## 1 Introduction

The prevalence of age-related memory declines and age-related neurodegenerative diseases is likely to escalate in the years to come, in pace with the rapid aging of the population. Exercise attenuates the symptoms of neurodegenerative diseases, such as Alzheimer and Parkinson [1]. The positive changes observed in the brain after exercise are partly mediated by the induction of BDNF and VEGF expression in the hippocampus [2]. However, the molecular pathways responsible for exercise mediated BDNF and VEGF induction have been elusive. Identifying molecules produced during exercise that mediate learning and memory formation will allow us to harness the therapeutic potential of exercise.

In recent years, an increased understanding of lactate has suggested a close relationship between lactate and brain function [[2], [3], [4]]. As early as 2013, Leiz discovered that lactate administration reproduces specific brain and liver exercise-related changes [5]. Besides, Cecilie reported that exercise induces cerebral VEGF and angiogenesis via the lactate receptor, and this effect is HIF-1α independent [6]. In addition, Yaeli proposed that lactate partially mediates the effect of physical exercise on adult rodents' neurogenesis but without changes in cognition tests [7]. Furthermore, using inhibitors, Lauretta discovered that lactate mediates the effects of exercise on learning and memory through SIRT1/PGC1a/FNDC5/BDNF pathway. In this study, lactate intervention significantly increased adult rodents' performance in the Morris Maze test [8]. In recent years, Marvin noticed that lactate induces neurogenesis in the mouse ventricular-subventricular zone via the lactate receptor [9], and one study proposed that even acute exercise-induced lactate can mediate mitochondrial biogenesis in the hippocampus of mice [10]. Altogether, current knowledge has verified that exercise and its by-product, lactate, have positive effects on the brain function of rodents. However, there are still some unrecognized questions. Jun's study proposed that the dose of lactate concentrations is a critical factor in lactate's effects because chronic lactate accumulation damages the brain activity of rodents' brain [11]. Current lactate studies use a variety of lactate injection protocols, and the results are not in accordance. In addition to the dose-dependent effects of lactate, current lactate studies only use adult animals and lack a comparison with the exercise effect. To further explore these undetermined areas of lactate functions, this study first investigated the effects of different doses of lactate intervention on brain neuroplasticity of aging mice (20–22months) to select the best intervention protocol. And then use this concentration to aged mice (25–27months) to check the effects of long-term lactate intervention and HIIT on brain neuroplasticity in aged rodents, thereby provides insight for potential clinical uses of lactate on age-related neurodegenerative symptoms.

## 2 Method

### 2.1 Grouping

Experiment 1: twenty 20–22 months old wild-type SPF female mice (C57BL/6J, The Jackson Laboratory) were randomly divided into five groups (n = 4), including 1. Control group, which receives PBS injections. 2. High-intensity interval training group. 3. High-dose lactate injection group. 4. Medium-dose lactate injection group 5. Low-dose lactate injection group.

Experiment 2：twenty-one 25–27 months old wild-type SPF male mice (C57BL/6J, The Jackson Laboratory) were randomly divided into three groups (n = 7) including 1. Control group, which receives PBS injections. 2. High-intensity interval training group. 3. Low-dose lactate injection group.

During the experiment, all animals were maintained in a thermoneutral room on a 12:12 h photoperiod and provided standard laboratory chow (VRF1 autoclavable rodent diet; DS801909G10R) and water ad libitum. All experiments were approved by the animal ethics committee of Sichuan University (K2020004) and conform with all the applicable institutional and governmental regulations on the ethical use of animals.

### 2.2 Lactate intervention protocol

High-dose lactate group mice received 2000 mg lactate per 1 kg body weight of sodium lactate (Sigma-Aldrich, 71718) dissolved in phosphate-buffered saline (PBS; Sigma, 806552)-pH7.4, sterile-filtered. Medium-dose lactate group received 1000 mg/kg of sodium lactate in PBS. Low-dose lactate group received 500 mg/kg of sodium lactate in PBS. The control for the lactate groups, the PBS group, and the HIIT groups were administered PBS at a volume that approximated the average volume administered to the lactate mice, around 0.2–0.3 ml per animal. Injections were performed intraperitoneally five times per week for 7 weeks in experiment1 and 6 weeks in experiment2 (Fig. 7). Two days after the intervention, all animals were anesthetized with intraperitoneal injections of ketamine (50 mg/kg; University of Veterinary Medicine Budapest) perfused by saline. The brain was quickly removed, weighed, and the hippocampus was dissected, frozen in liquid nitrogen, and stored at −80 °C degree. A section of the hippocampus was homogenized in a buffer containing: 137 mM NaCl, 20 mM Tris-HCl pH8.0, 2 % NP 40, 10 % glycerol, and protease inhibitors (PMSF, aprotinin, leupeptin, orthovanadate). Protein levels were determined using Bradford techniques (Bio-Rad).

### 2.3 Training protocol

Mice from HIIT groups first participated in 3 days of adaptation training on a motorized treadmill to limit training stress. After the adaptation, mice in the HIIT group underwent a modified incremental speed test at a 10 % incline [6]. The test includes a 5 min warm-up at 6 m/min and then gradually increases the speed for 1 m/min every 2 min until the mice cannot maintain the original movement speed on the running machine and resist moving after stimulation for 10s. We removed those who refused to run and recorded the speed. Eventually, the average max speed is used to calculate the HIIT protocol speed in the formal test. The average max-speed in the aging mice group (20–22 months) is 22 m/min, while the max-speed in the aged mice group (25–27 months) is 18 m/min. In the formal test, the high-intensity interval training consists of 3min of 85 % max speed and 2 min of 45 % max speed for ten cycles at a 10 % incline, starting with a 5min warm-up at 50 % max speed [6]. The training was conducted five days per week for 7 weeks in experiment 1 and 6 weeks in experiment 2.

### 2.4 Open field tests

The open-field test is widely used to study the reaction to novelty and provides some insight into the state of anxiety in rodents [12]. Mice were positioned in the center of an open-field box consisting of a cylindrical arena of 80 cm diameter, divided into 20 sectors by concentric and radial lines, and surrounded by a 35 cm-high wall. The exploration time for each mouse is 5 min. The video is recorded to evaluate the time mice spent in inner and outer zones, which is widely used to indicate the animals' stress level and exploration of novelty.

### 2.5 Novel object recognition tests

The novel object recognition test is used to study rodents' memory functions [13]. This test was performed in an open-field box (62.5x34.5 × 32 cm). Three trials were conducted per session. In the first trial, each mouse is placed in the middle of the empty open area and allowed for free exploration for 5 min. After 24 h, each mouse was placed in the same area with two identical objects in the opposite quadrants of the area (NE and SW corners) and allowed free exploration for 5 min. The last test is conducted 3 h after the second trial. A new object with a different size, color, shape, and material replaced the one used in the second test. The object's location remained the same to avoid any influence of spatial memory. The box and objects were cleaned after each animal to remove any scent. The interaction time was recorded with the object (e.g., touching, climbing, and sniffing with the nose at a 2 cm distance). Memory's recognition index was calculated by the formula: time to investigate the new object/time to investigate both objects.

### 2.6 Passive avoidance tests

The Passive Avoidance task is a fear-aggravated test used to evaluate learning and memory in rodent models [14]. The apparatus comprised two equally sized compartments, a dark one and a well-lit white compartment (20 × 25 × 25 cm each), separated by a small sliding door. All mice were brought to the testing room at least 30 min before testing, and the lights were turned off to allow the animals to acclimate to the darkened room. For the experiment, each mouse was picked up gently by its tail, removed from the home cage, and placed onto the grid floor in the illuminated area of the box, facing away from the door. The timer was initiated when the mouse was placed into the chamber. After 5 s, the door to the darkened area of the chamber was opened. Once the mouse entered the darkened area of the chamber, the door was closed, and scrambled footshock (0.8 mA) was delivered for 2 s. The animal remains in the dark compartment for an additional 20 s after terminating the aversive stimulus before being removed and placed back into its home cage. After 24 h, the test was conducted again, and the latency of the entrance into the dark compartment was recorded. If a mouse failed to walk into the dark chamber within 300 s, then the timer was stopped, a maximum 300-s latency was recorded, and the mouse was returned to its home cage.

### 2.7 Western blot

Proteins were electrophoresed on 8–12 % v/v polyacrylamide SDS-PAGE gels and were transferred onto PVDF membranes. The membranes were subsequently washed, and after blocking, PVDF membranes were incubated at 4 °C with antibodies including Tubulin (T6199), β-Actin (sc69879), VEGF (sc152),p-MTOR/MTOR (cst5536,2983), p-AKT/AKT (cst9271,4691), nNOS (bd610309), eNOS (ab76198), iNOS(cst13120), SIRT3 (cst#2627), Pan-Kla (STJ11101522), HIF-1α (H6536), BDNF (sc546), PGC-1α (nbp1-04676), SIRT1 (ab110304), CS (ab96600), SDHA (sc98253), FNDC5 (ab174833), *p*-HSL/HSL (ma5-35896, cst18381s),p-CREB/CREB (cst9198,9197s), LDH (sc33781), Visfatin/Nampt (ab45890), and Synapsin (cst2312). After incubation with primary antibodies, membranes were washed 3 × 10 min in TBS-Tween-20 (TBS-T) and incubated with horseradish peroxidase (HRP)–conjugated secondary antibodies (Jackson Immunoresearch) at 4 °C for 1 h. After incubation with a secondary antibody, membranes were repeatedly washed and developed by HRP reagent (Super Signal West Pico Chemiluminescent Substrate, Thermo Scientific #34080). The bands were quantified by ImageJ software and normalized to beta-actin or tubulin, which served as an internal control. For all the Phosphorylated proteins, the phosphorylated one is developed at first, and then the same PVDF was striped and repeated for the first antibody of the total protein. The ratio is calculated based on the protein expression from the same PVDF.

### 2.8 Blood lactate measurement

Portable electrochemical devices Lactate Scout and Sensors (nova biomedical,40828/40813) were used to determine the blood lactate levels in a previously described protocol [15]. A drop of blood was excised from the aseptically treated wound of the mice's tail. After resting for 20 min, resting blood lactate concentration was determined. Then the mice were placed in a separate cage to avoid external stimuli for the subsequent tests. After different doses of lactate injection, the blood lactate level is tested at several time points until the blood lactate level recovers to resting concentration.

### 2.9 Tissue lactate and pyruvate concentration measurement

Lactate and pyruvate concentration of the hemisphere after long-term exercise and lactate intervention was assayed using pyruvate and lactate assay kits (Abcam; ab65330, ab65342). About 40 ± 2 mg of the homogenized left hemisphere of seven mice of each group were weighed and lysed on ice. After deproteination, the sample was analyzed according to the protocol provided by the kit.

### 2.10 Statistical analysis

The statistical analysis was performed using one-way analysis of variance (ANOVA) analysis followed by Dunnett's multiple comparisons tests using Graph pad prism version 9.1 software. Data were reported as mean ± standard deviation (SD). Statistical significance was denoted as *p＜0.05.

## 3 Result

### 3.1 The dose-dependent effects of lactate, and exercise on brain function of aging mice

**Fig. 1:**
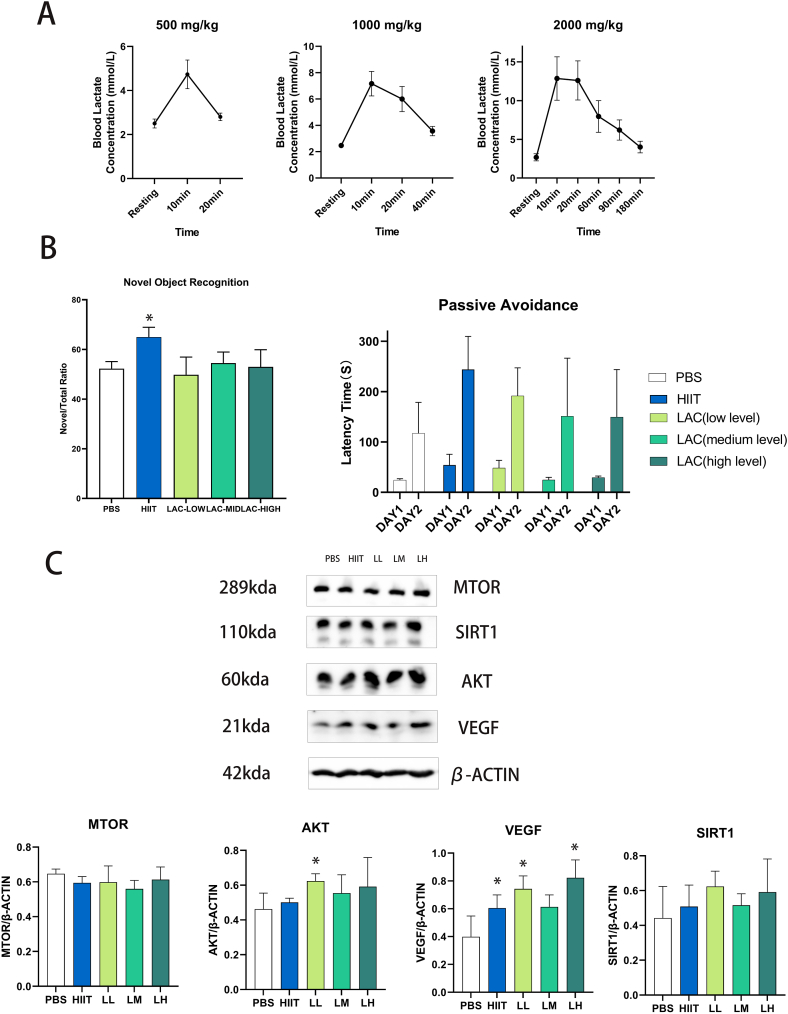
(A) The effect of different doses of lactate interventions on blood lactate levels. (B) The effect of different doses of lactate injections, and exercise on cognition tests performance of aging mice. (C) The effects of lactate and exercise on brain plastic biomarkers of aging mice's hippocampus. Data are presented as means ± SD (n = 4). Western blot data is based on each animal's protein expression. Low-level lactate injection (LL); Medium-level lactate injection (LM); High-level lactate injection (LH). The statistical analysis was performed using one-way analysis of variance (ANOVA) analysis followed by Dunnett's multiple comparisons. Statistical significance was denoted as *p＜0.05. The Original uncropped pictures are provided in the supplementary material.

The primary aim of the 1st experiment is to test the dose-dependent effect of lactate and select the best volume to conduct the formal experiment. Three days before the start of the intervention, we confirmed the effects of one-time lactate injection on blood lactate concentration. As shown in Fig. 1(A), the peak blood lactate concentration of the low-level lactate group reached 4.73 ± 0.65 mmol/L 10 min after one-time lactate injection and decreased to baseline levels at 20 min following injection. In the medium-level lactate group, peak lactate concentration reached 7.16 ± 0.93 mmol/L 10 min after injection and decreased to baseline levels at 40 min following injection. As for the high-level lactate group, peak lactate concentration reached 13.85 ± 2.8 mmol/L 10 min after intervention and decreased to baseline levels at 180 min following injection. In summary, higher injection volume will increase the blood lactate level and the recovery time. After five weeks of intervention, we tested the animal's performance in the novel object recognition and passive avoidance tests. As shown in Fig. 1(B), five weeks of HIIT significantly increased (p = 0.014) the novel/total ratio in the novel object recognition test compared with the control group. While low, medium and high-level lactate injection does not affect novel object recognition tests. Furthermore, five weeks of HIIT (p = 0.0949) and low-level lactate injection (p = 0.416) slightly increased the latency time in the passive avoidance test, but the result is insignificant. After seven weeks of intervention, we examined several neuro-plastic biomarkers in the hippocampus, including SIRT1, VEGF, MTOR and AKT, as indicated in Fig. 1(C). The result shows that both low (p = 0.009) and high doses (p = 0.034) of lactate injection significantly increase the protein expression of VEGF compared with the control group. In addition, SIRT1 and AKT are slightly increased in the low and high lactate injection groups; however, only low-level lactate injection (p = 0.043) significantly increased AKT, while the others are insignificant. Besides, we noticed that the animals' movement is slowed immediately after the high-dose lactate injection, and they behaved lethargy, including being immobile and staying in the corner. We hypothesized that 13.85 ± 2.8 mmol/L blood lactate concentration might cause animal discomfort. Altogether, this experiment verified that both low and high-dose lactate positively affect the brain function of aging mice. Since the low-dose protocol is comparatively safer and as effective as the high dose, we used the low-dose lactate protocol in subsequent experiment.

### 3.2 The effect of lactate and exercise on cognition tests performance of aged mice

**Fig. 2:**
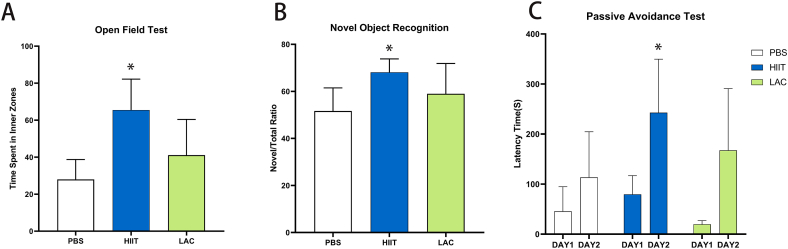
(A) Open field test (B) Novel object recognition test (C) Passive avoidance test. Data are presented as means ± SD (n = 7). The statistical analysis was performed using one-way analysis of variance (ANOVA) analysis followed by Dunnett's multiple comparisons. Statistical significance was denoted as *p＜0.05.

In the 2nd experiment, after five weeks of intervention, animals in all groups underwent cognition tests in a sequence of open-field tests, novel object tests, and passive avoidance tests. As shown in Fig. 2 (A), compared with the control group, HIIT significantly increased (p＜0.001) the time (S) the animal spent in the inner area, while lactate did not affect (p = 0.263) the performance of aged mice in the open field test. Fig. 2 (B) indicates that five weeks of HIIT significantly increased (p = 0.021) the investigation time of the novel object compared to the control group, while lactate slightly increased (p = 0.397) the ratio, but the result is insignificant. In the end, Fig. 2 (C) indicate that HIIT significantly increased (p = 0.044) the latency time the animal entered the dark chamber, while lactate did not affect (p = 0.599) the aged mice's performance on the passive avoidance test. These results show that five weeks of HIIT effectively improves aged mice's performance in the open field, novel object recognition, and passive avoidance tests. Conversely, five weeks of lactate injection does not affect these tests.

### 3.3 The effect of lactate and exercise on hippocampus angiogenesis signaling pathway in aged mice

**Fig. 3:**
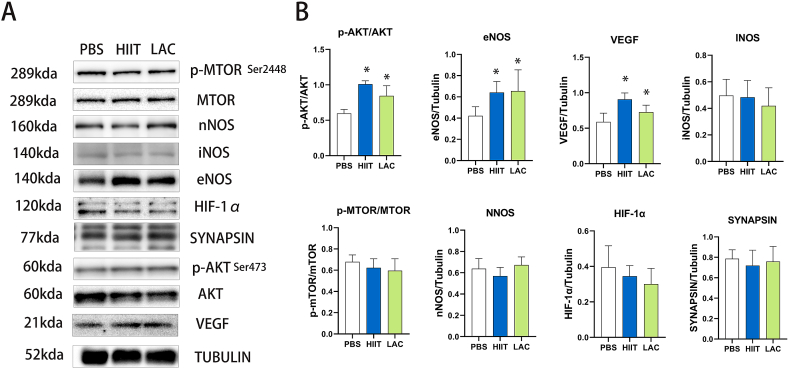
The effect of lactate and HIIT on hippocampus angiogenesis signaling pathway in aged mice. (A) Western blot pictures (B) Statistical analysis. Data are presented as means ± SD (n = 5). The statistical analysis was performed using one-way analysis of variance (ANOVA) analysis followed by Dunnett's multiple comparisons. Statistical significance was denoted as *p＜0.05. The Western blot statistics are calculated by each animal's data, and the highest and lowest in each group are excluded from the statistic to remove extreme data.

As shown in Fig. 3, compared with the control group, after six weeks of intervention, both HIIT (p＜0.001) and lactate injection (p = 0.002) significantly increased the activity of AKT signaling (ser473), indicating elevated angiogenesis in the hippocampus of aged mice. However, the activity of its downstream signal, MTOR (ser2448), has not changed after the intervention. As a result of the elevated AKT pathway, the ENOS protein expression is significantly increased in both HIIT (p = 0.048) and lactate (p = 0.042) groups. Furthermore, the protein expression of VEGF is also significantly increased in the HIIT (p＜0.001) and lactate group (p = 0.045). In addition, we also tested several brain angiogenesis-related proteins, including NNOS and HIF-1α. Our result discovered that exercise and lactate do not affect the protein expression of NNOS and HIF-1α in the hippocampus of aged mice. Besides, our result shows that exercise and lactate do not affect the protein expression of SYNAPSIN.

### 3.4 The effect of lactate and exercise on hippocampus BDNF signaling and mitochondrial biomarkers in aged mice

**Fig. 4:**
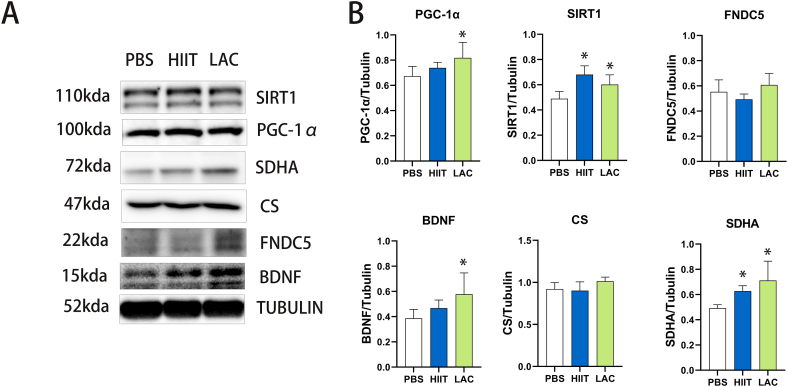
The effect of lactate and HIIT on hippocampus BDNF signaling and mitochondrial biomarkers in aged mice. (A) Western blot pictures (B) Statistical analysis. Data are presented as means ± SD (n = 5). The statistical analysis was performed using one-way analysis of variance (ANOVA) analysis followed by Dunnett's multiple comparisons. Statistical significance was denoted as *p＜0.05. The Western blot statistic is calculated by each animal's data, and the highest and lowest in each group are excluded from the statistic to remove extreme data.

Recent lactate and exercise studies have proposed that the elevation of BDNF is achieved through the PGC-1α-SIRT1-FNDC5-BDNF signaling [8]. As shown in Fig. 4, compared with the control group, lactate significantly increased (p = 0.035) the BDNF protein expression in the hippocampus of aged mice. The upper signaling proteins, including PGC-1α (p = 0.04), SIRT1 (p = 0.034), and FNDC (p = 0.226), also show an increasing tendency. On the contrary, the effect of HIIT on BDNF signaling in the hippocampus of aged mice is minimal. Six weeks of HIIT slightly increased the protein expression of BDNF (p = 0.407) and PGC-1α (p = 0.403); however, the result is insignificant. Only the SIRT1 protein expression is significantly elevated (p＜0.001) by HIIT. Furthermore, we also involved two mitochondrial biomarkers, CS and SDHA. Our result shows that both HIIT (p = 0.047) and lactate (p = 0.005) significantly increased the protein expression of SDHA but without the increase of CS (HIIT p = 0.866; Lactate p = 0.076).

### 3.5 The effect of lactate and exercise on metabolic content and related signaling in aged mice

**Fig. 5:**
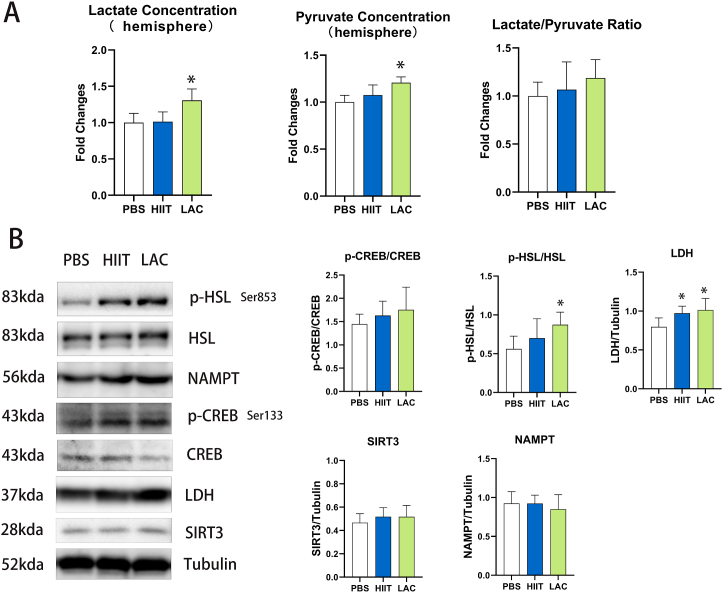
(A) The effect of chronic lactate and exercise interventions on pyruvate and lactate concentration in the hemisphere of aged mice. (B) The effect of lactate and HIIT on hippocampus metabolism-related signaling in aged mice. Data are presented as means ± SD (n = 5). The statistical analysis was performed using one-way analysis of variance (ANOVA) analysis followed by Dunnett's multiple comparisons. Statistical significance was denoted as *p＜0.05. The Western blot statistic is calculated by each animal's data, and the highest and lowest in each group are excluded from the statistic to remove extreme data.

Recent studies have proposed that lactate is crucial in regulating energy metabolism [16]. However, whether lactate affects metabolic pathways in the brain of aged mice is still unclear. In Fig. 5 (A), compared with the control group, the lactate group shows a significant increase in pyruvate and lactate levels in the hemisphere of aged mice. However, the lactate/pyruvate ratio does not change after the intervention. As presented in Fig. 5 (B), chronic HIIT slightly increased the signaling pathway of CREB (p = 0.542) and HSL (p = 0.356) but without significant changes. On the other hand, lactate induced more prominent elevation. Six weeks of lactate intervention significantly increased (p = 0.047) the HSL activity but failed to elevate (p = 0.225) pCREB/CREB protein expression. Furthermore, both HIIT (p = 0.037) and lactate (p = 0.009) significantly elevated LDH protein expression while does not affect the protein expression of SIRT3 and NAMPT.

### 3.6 The effect of lactate and exercise on Pan Lactic acid-Lysine protein expression

**Fig. 6:**
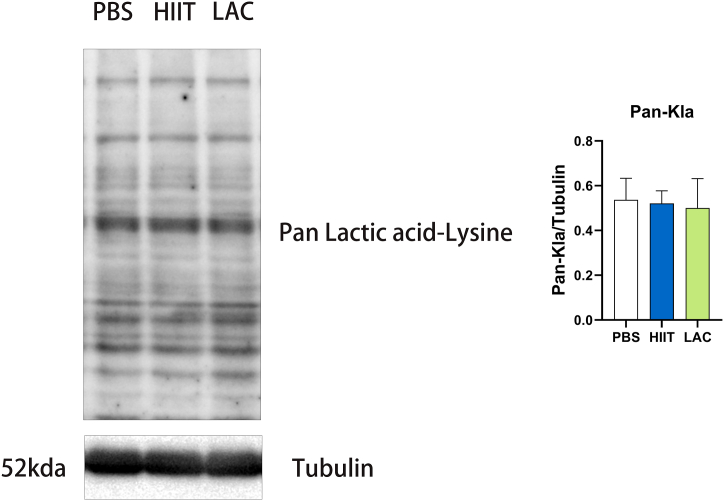
The effect of lactate and HIIT on Pan Lactic acid-Lysine protein expression in aged mice. Data are presented as means ± SD (n = 5). The statistical analysis was performed using one-way analysis of variance (ANOVA) analysis followed by Dunnett's multiple comparisons. Statistical significance was denoted as *p＜0.05. The Western blot statistics are calculated by each animal's data, and the highest and lowest in each group are excluded from the statistic to remove extreme data.

Studies have proposed that lactate-derived lactylation of histone lysine residues serves as an epigenetic modification that directly stimulates gene transcription [17]. In Fig. 6, our result shows that chronic lactate and HIIT intervention does not affect the Pan Lactic acid-Lysine protein expression in the hippocampus of aged mice.

**Fig. 7:**
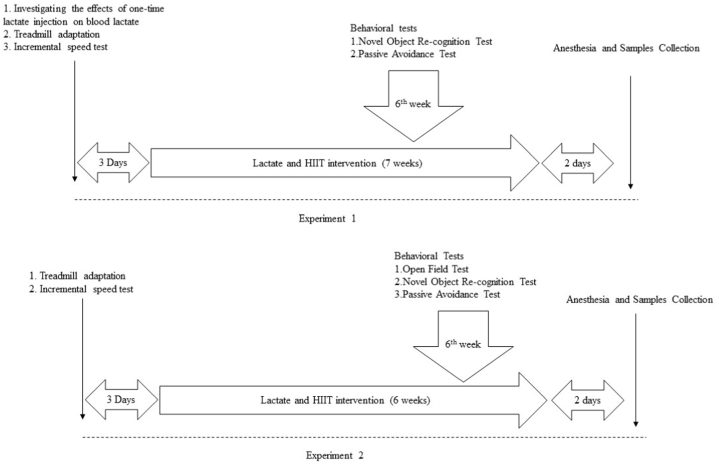
Timeline of the experiment.

## 4 Discussion

This is the first study investigating the dose-dependent effects of lactate on brain functions of aging rodents, as well as exploring the impact of lactate on aged rodents’ brain function. In the 1st experiment, our result demonstrates that both high (2000 mg/kg) and low (500 mg/kg) doses of chronic lactate injection have a positive influence on brain function biomarkers, including SIRT1, VEGF, and AKT in aging mice. However, no changes were observed in behavior tests. Conversely, long-term HIIT significantly improves performance in novel-object recognition tests for aging mice.

In the 2nd experiment, our finding reveal that long-term lactate treatment (500 mg/kg) has beneficial effects on angiogenesis signaling pathway (p/t-AKT, eNOS and VEGF), BDNF signaling (PGC-1α, SIRT1 and BDNF), mitochondrial biomarkers (SDHA), metabolism-related proteins (p/t-HSL and LDH) in the hippocampus of aged mice. However, chronic lactate treatment does not improve aged mice's performance in the open field, novel-object recognition tests, and passive avoidance tests. Conversely, chronic HIIT significantly improved aging mice's performance in these behavior tests. In summary, the present study proved that chronic HIIT and lactate treatment positively impact the brain functions of aged mice.

Research to date has provided novel insights into lactate's positive role in multiple brain functions and several brain diseases. Reviews from Cai, Hashimoto, and Muller have systematically summarized the function of lactate in the brain [[2], [3], [4]]. Based on current knowledge, this study tries to provide evidence of the dose-dependent effect of lactate and the effect of lactate on aged rodents. In the 1st experiment, we selected three doses of lactate concentration: 1.2000 mg/kg resulting in the blood lactate level reaching 13.85 ± 2.8 mmol/L 10 min after intervention and decreasing to baseline levels at baseline 180 min following injection. 2.1000 mg/kg resulting in the blood lactate level reaching 7.16 ± 0.93 mmol/L 10 min after injection and decreasing to baseline levels at 40 min following injection.3.500 mg/kg resulting in the blood lactate level reaching 4.73 ± 0.65 mmol/L 10 min after lactate injection and decreasing to baseline levels at 20 min following injection. In summary, our results show that the effect of lactate injection on blood lactate concentration is dose dependent. Higher injection volume will increase the blood lactate level and the recovery time. Our data is in accordance with the previous studies. Cecilia's study [6] discovered that 2500 mg/kg level of lactate injection elevates the blood lactate concentration to 14 mM, and after 7 weeks of intervention, the cerebral VEGF expression and angiogenesis are increased in adult rodents. Yaeli's study [7] suggested that a 1750 mg/kg level of lactate injection elevated the blood lactate level to 15.2 ± 1.94 mM and 2 weeks after the intervention, hippocampal neurogenesis is increased in adult rodent. Lauretta's study [8] proposed that 180 mg/kg and 117 mg/kg lactate injections can increase hippocampal lactate concentration, and after lactate injection, the BDNF signaling is activated in adult rodents. Here, this study concluded previous injection protocols and made a comparison of the dose-dependent effect of lactate. Our result shows that both low and high-dose lactate have the same effects on biomarkers of brain function. Interestingly, the middle dose of lactate injection is less effective than the low and high doses. Because of our limited animal number, we cannot verify but further hypothesize that the dose of lactate intervention is a critical factor in lactate's effects. As a result, we used the low-level lactate injection protocol for the following up formal study. It is also important to mention that after different doses of chronic lactate intervention, the aging animals' performance in novel object recognition test and passive avoidance test did not change. On the contrary, chronic HIIT significantly increased the performance of aging mice in novel object recognition test and passive avoidance test.

Current research has well-established that exercise delays brain aging, preserves memory and cognition, and improves symptoms of neurodegenerative pathologies [1]. In the 2nd experiment, we verified that long-term HIIT has a positive effect on the brain function of aged mice, indicated by the significant increase in the performance in the open-field test, novel object recognition test, and passive avoidance test. This result is in line with previous studies. Several experiments have discovered that long-term exercise can increase the aged mice's performance in passive avoidance tests, novel object recognition tests, and open field tests [18,19]. Regarding lactate's effect, our data show that lactate intervention alone can slightly increase the mice's performance in the passive avoidance test and novel object recognition test, but the result is not significant. According to previous studies conducted on adult animals, some researchers proposed that lactate can increase the mice's performance in the Morris maze test [19], while other studies suggest that lactate cannot increase the performance [7]. Our results prove that lactate alone is less effective than HIIT in improving aged mice's learning and memory ability and exploration habits, as indicated by behavior tests.

In addition to cognition tests, this study also investigated the brain function-related signaling pathway. Numerous studies have concluded that exercise and lactate intervention can increase the angiogenesis signaling pathway in adult rodents, and the brain's angiogenesis is closely related to brain function [6]. However, whether lactate affects aged animals is still unclear. Investigating this question will provide evidence for the clinical usage of lactate in neurodegenerative diseases. Our results show that both chronic HIIT and lactate intervention significantly increased the activity of AKT signaling, indicating elevated angiogenesis in the hippocampus of aged mice [20]. However, the downstream signal, MTOR, has not changed after the intervention. As a result of the elevated AKT pathway, the eNOS and VEGF protein expression is significantly increased in both HIIT and lactate groups. In addition, we also tested several brain angiogenesis-related proteins, including nNOS, iNOS and HIF-1α. Our result discovered that exercise and lactate do not affect the protein expression of these proteins in the hippocampus of aged mice. Altogether, our results show that both long-term exercise and lactate intervention can activate hippocampus angiogenesis in aged mice.

Furthermore, this study investigated the effect of lactate and exercise on BDNF signaling and mitochondrial biomarkers, including PGC-1α, SIRT1, FNDC5, BDNF, CS, and SDHA. As early as 2013, Christiane's study [21] discovered that exercise induces hippocampal BDNF through a PGC-1α/FNDC5 pathway, and the elevation of BDNF is associated with improved cognitive function. In recent years, Lauretta [8] proposed that lactate promotes BDNF through PGC-1α, SIRT1, FNDC5, and BDNF signaling. Also, studies have suggested that lactate intervention has a positive effect on mitochondrial in the brain [10], skeletal muscle [15,22], and white adipose tissues [23]. However, data regarding aged rodents is unclear. Our results show that lactate significantly increased the BDNF protein expression in the hippocampus of aged mice. The upper signaling protein, including PGC-1α, SIRT1, and FNDC, also shows an increasing tendency. On the contrary, the effect of HIIT on BDNF signaling in the hippocampus of aged mice is minimal. Six weeks of HIIT slightly increased the protein expression of BDNF and PGC-1α; however, the result is insignificant. Only the SIRT1 protein expression is significantly elevated by HIIT. Previous studies also reported that the effect of exercise on BDNF expression is smaller in aged animals compared to young ones [24]. Here, we prove that long-term lactate intervention is more effective than exercise in promoting BDNF signaling. Furthermore, we also involved two mitochondrial biomarkers, CS and SDHA. Our result shows that both HIIT and lactate significantly increased the protein expression of SDHA but without the increase of CS. Altogether, our results show that lactate effectively improves BDNF signaling and mitochondrial biomarkers in the hippocampus of aged mice, and this effect is more substantial than exercise.

Recent studies have suggested a close relationship between lactate and metabolism. Joshua's study proposed the underestimated physiological potential of lactate [25]. However, whether long-term lactate intervention affects metabolic pathways in the brain of aged mice is still unclear. Previous study has reported that the increased lactate level in the brain is related to the increased brain blood flow [26]. On the contrary, lactate content in the brain decreases during memory impairment in mouse models of AD [27]. Furthermore, recent studies have proposed that pyruvate, in addition to its well-recognized function in energy metabolism, has a unique combination of neuroprotective effects [28]. As presented in this study, our data suggest that long-term lactate intervention significantly increased the lactate and pyruvate concentration in the hemisphere of aged mice without changing the lactate and pyruvate ratio. While compared with the control group, long-term HIIT does not alter the pyruvate and lactate concentration. Brain LDH is responsible for the interconversion of pyruvate and lactate. Previous studies have reported that blood LDH levels can be triggered by both resistance and aerobic exercise [29]. To our knowledge, this is the first study examining the effect of HIIT and lactate intervention on aged rodents' hippocampus LDH expression. Our results show that chronic lactate and HIIT can elevate LDH and thereby may positively improve the capacity to process lactate and pyruvate. Also, our results show that chronic HIIT slightly increased the signaling pathway of CREB/HSL but without significant changes. On the other hand, lactate induced more prominent elevation. Long-term lactate intervention significantly increased the HSL activity with nonsignificant elevated p/t-CREB/CREB expression. Previous studies have indicated that the activation of CREB can promote BDNF expression [30]. In our study, long-term lactate intervention slightly increased CREB activity with elevated BDNF protein expression. As a result of CREB elevation, the phosphorylation of the HSL ratio also increased. The functional role of this enzyme in the brain remains unexplored. This study first proposes that lactate intervention can significantly increase the activity of HSL in a way that might be related to CREB. Moreover, this study reports that HIIT and lactate do not affect the protein expression of SIRT3 in the hippocampus of aged mice. Radak's study [31] has indicated that the effect of exercise on SIRT3 expression is time-dependent; thus, the duration of our training protocol is not enough to trigger SIRT3 elevation. While NAMPT/VISFATIN is the rate-limiting enzyme in NAD + salvage pathways [32]. Our data show that chronic exercise and lactate intervention does not change the protein expression of NAMPT/VISFATIN. Altogether, our results show that lactate is effective in elevating the pyruvate and lactate concentration, CREB/HSL signaling pathway and LDH protein expression in the brain of aged mice.

Recent studies have proposed that lactate-derived lactylation of histone lysine residues serves as an epigenetic modification that directly stimulates gene transcription [17]. Several studies have identified the pivotal role of protein lactylation in cell fate determination, embryonic development, inflammation, cancer, and neuropsychiatric disorders, offering key breakpoints for further functional and mechanistic research [33]. Our result shows that lactate and HIIT intervention does not affect the Pan Lactic acid-Lysine protein expression in the hippocampus of aged mice. It is important to mention that we harvested the sample 48 h after the last intervention. Further time-dependent and age-dependent studies are needed to further explore the mechanisms of lactate-induced lactylation.

In summary, our study demonstrates that long-term exercise and lactate interventions have a beneficial impact on brain function in aging and aged rodents. Specifically, the exercise group exhibits superior performance in cognition tests, upregulated angiogenesis signaling, and improved mitochondrial biomarkers compared to the control group. On the other hand, lactate intervention improves angiogenesis signaling, BDNF signaling, mitochondrial biomarkers, and metabolic content and signaling in the hippocampus of aged mice. Considering current knowledge and data from this study, we firmly suggest the potential utilization of lactate as a therapeutic strategy in neurodegenerative diseases.

Limitation: While this study provides insights into the effects of long-term lactate and high-intensity interval training (HIIT) on brain neuroplasticity in aged mice, there are several limitations that should be acknowledged: 1. The sample size in this study was limited. 2. Although neuroplasticity biomarkers were assessed, the study did not correlate these changes with specific behavioral outcomes. 3. The study explored long-term interventions; however, the optimal duration and dosage of lactate and HIIT for maximizing neuroplasticity effects remain unclear. 4. While correlations between lactate, HIIT, and neuroplasticity biomarkers were observed, the study design did not establish causation. Mechanistic insights into the pathways through which lactate and HIIT influence neuroplasticity were not fully elucidated and warrant further investigation.

## Funding

ZR acknowledge support from the National Excellence Program (126823) and the Scientific Excellence Program, TKP2020-NKA-17 and TKP2021-EGA-37, at the Hungarian University of Sport Science, Innovation and Technology Ministry, Hungary.

## Data availability statement

Data will be made available on request.

## CRediT authorship contribution statement

**Zhou Lei:** Writing – review & editing, Writing – original draft, Methodology, Investigation, Formal analysis, Conceptualization. **Soroosh Mozaffaritabar:** Methodology. **Takuji Kawamura:** Methodology. **Atsuko Koike:** Methodology. **Attila Kolonics:** Methodology. **Johanna Kéringer:** Methodology. **Ricardo A. Pinho:** Writing – review & editing. **Jingquan Sun:** Resources, Conceptualization. **Ruonan Shangguan:** Resources. **Zsolt Radák:** Writing – review & editing, Writing – original draft, Supervision, Conceptualization.

## Declaration of competing interest

The authors declare the following financial interests/personal relationships which may be considered as potential competing interests:Zsolt Radak reports financial support was provided by Hungarian University of Sports Science. Zsolt Radak reports financial support was provided by Innovation and Technology Ministry, Hungary. If there are other authors, they declare that they have no known competing financial interests or personal relationships that could have appeared to influence the work reported in this paper.

## Figures and Tables

**figs1:**