# Supplementary material for: The effects of long-term lactate and high-intensity interval training (HIIT) on brain neuroplasticity of aged mice
Source: Heliyon. 2024 Jan 10;10(2):e24421. doi: 10.1016/j.heliyon.2024.e24421 (PMC10826720; doi:10.1016/j.heliyon.2024.e24421)

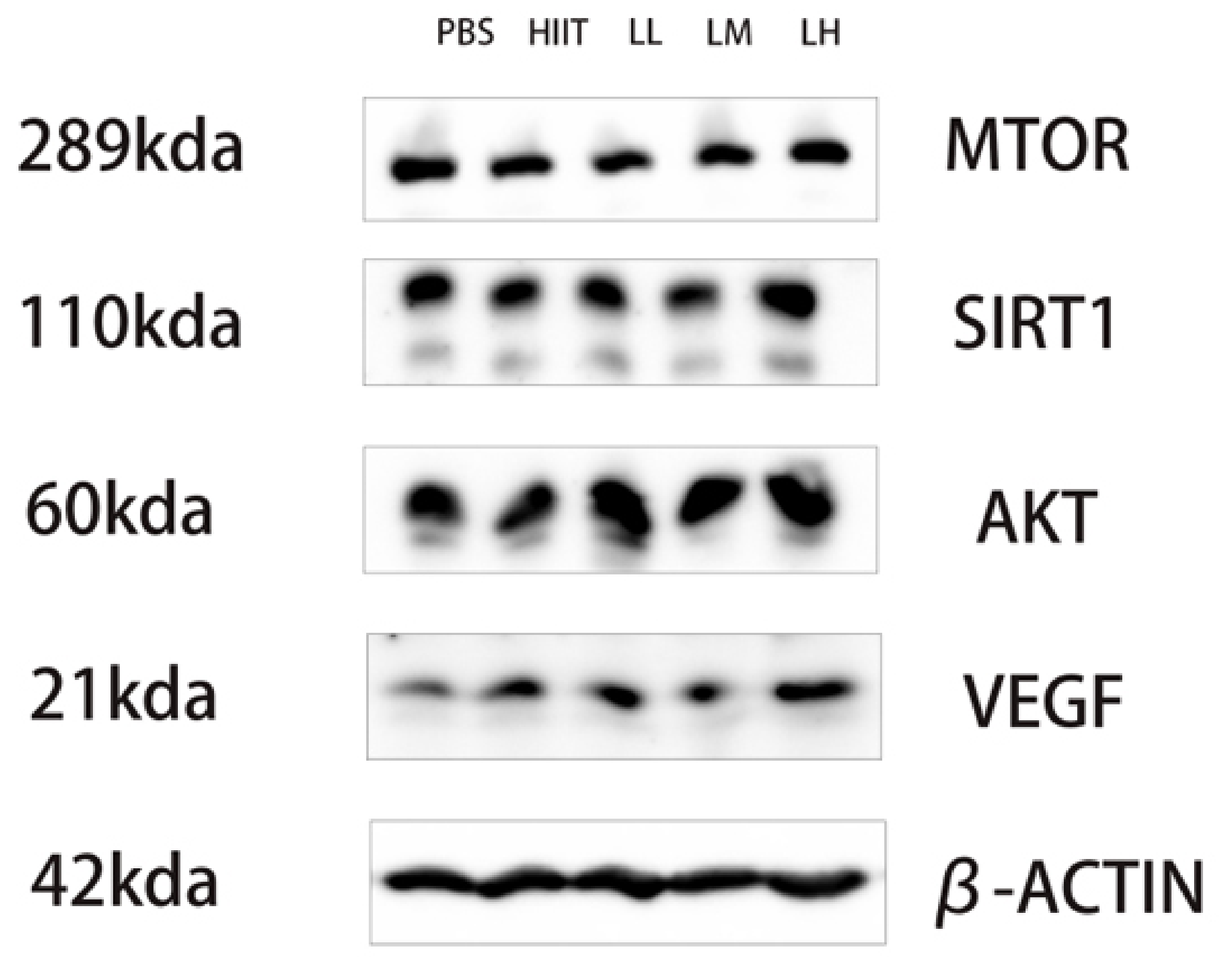
PBS HIIT LL LM LH
289kda MTOR
110kda SIRT1
60kda AKT
21kda VEGF
42kda β-ACTIN

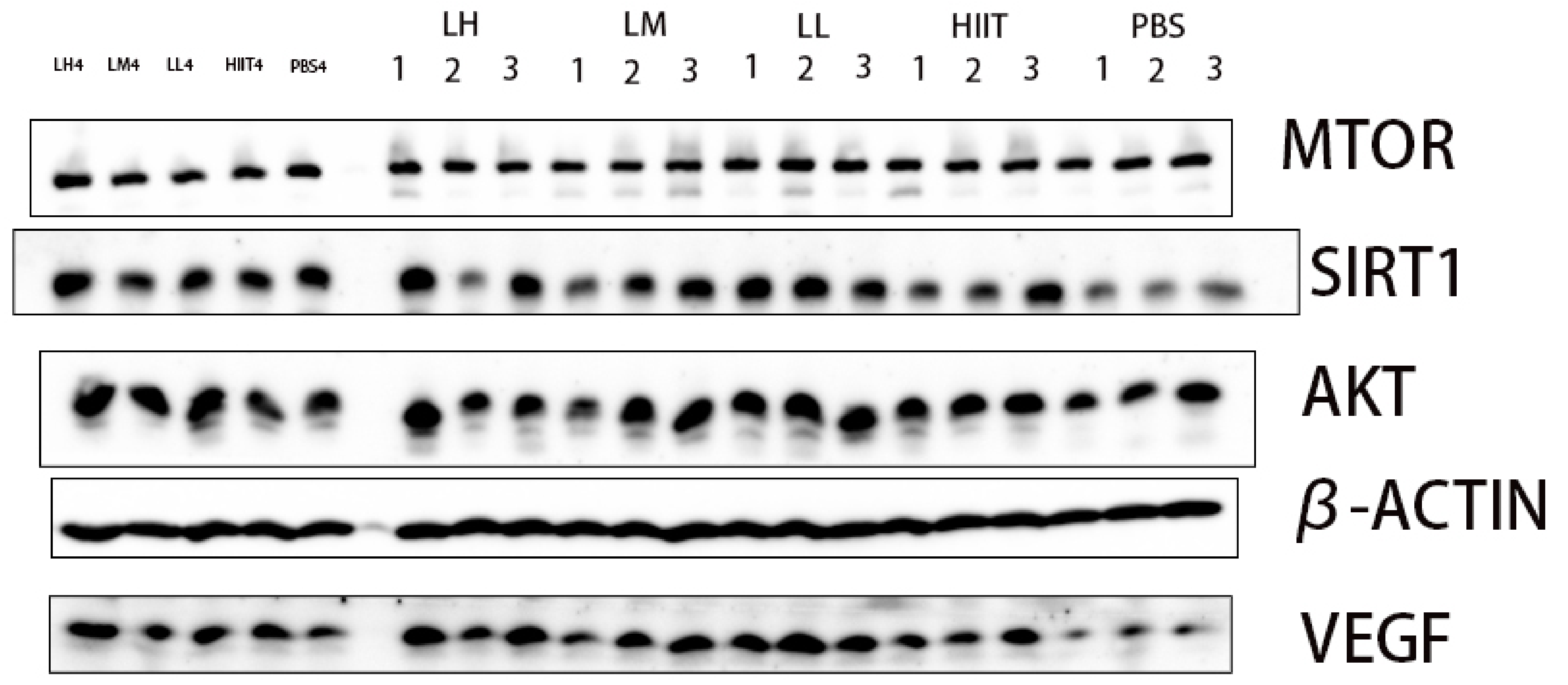
LH4 LM4 LL4 HIIT4 PBS4
LH 1 2 3
LM 1 2 3
LL 1 2 3
HIIT 1 2 3
PBS 1 2 3
MTOR
SIRT1
AKT
β-ACTIN
VEGF

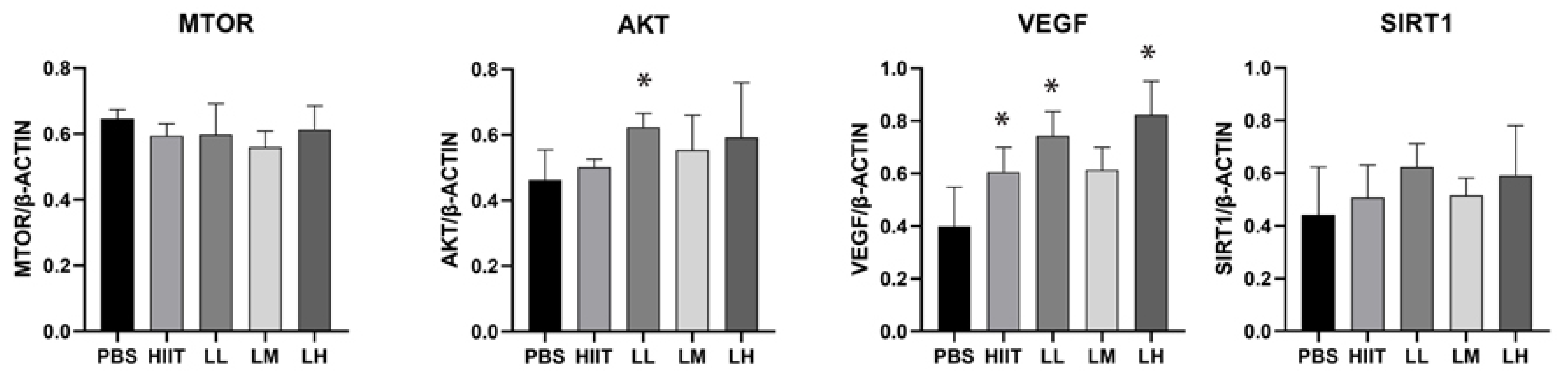
MTOR
MTOR/β-ACTIN
AKT
AKT/β-ACTIN
VEGF
VEGF/β-ACTIN
SIRT1
SIRT1/β-ACTIN
PBS HIIT LL LM LH

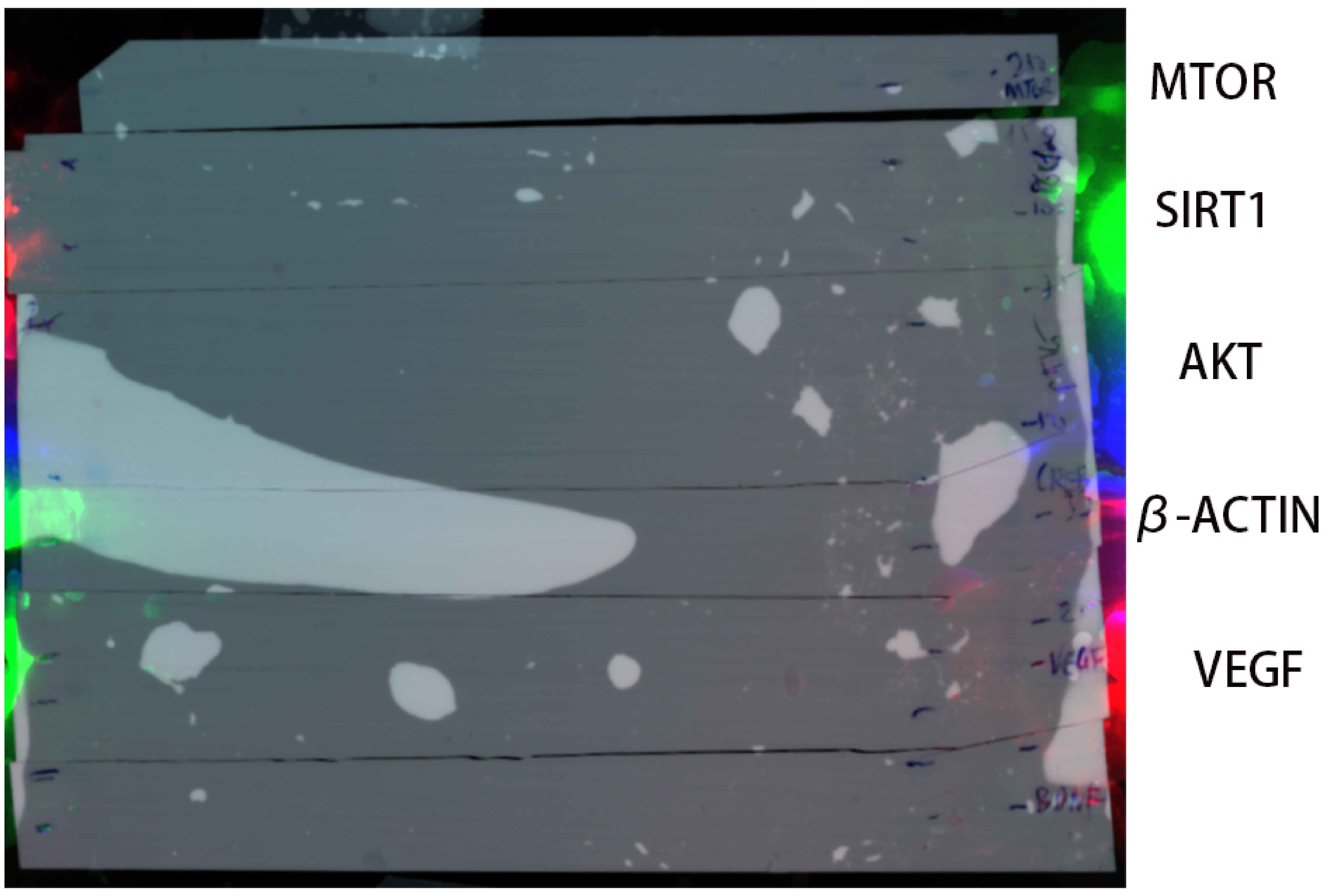
MTOR
SIRT1
AKT
β-ACTIN
VEGF

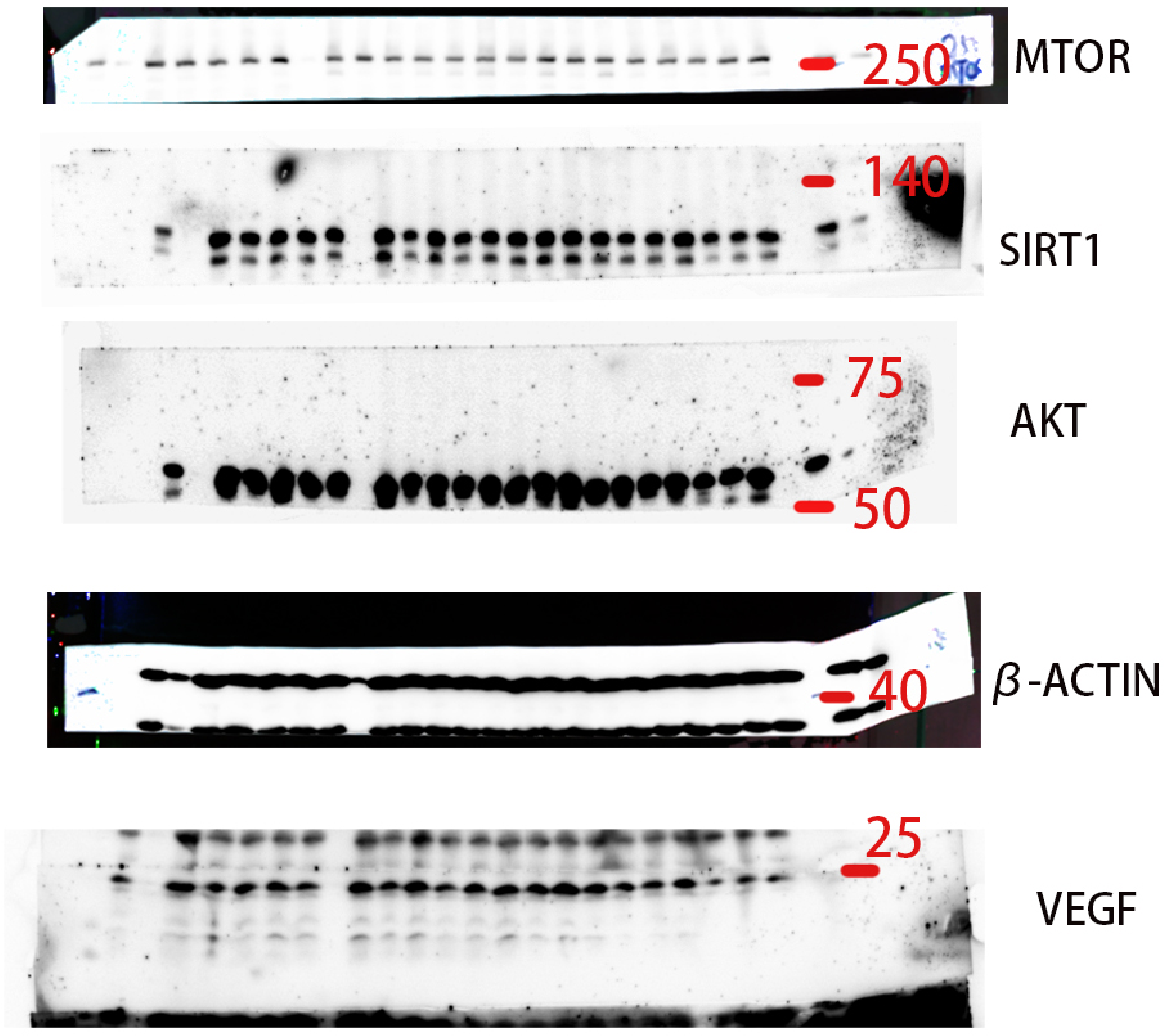
250
MTOR
140
SIRT1
75
50
AKT
40
β-ACTIN
25
VEGF

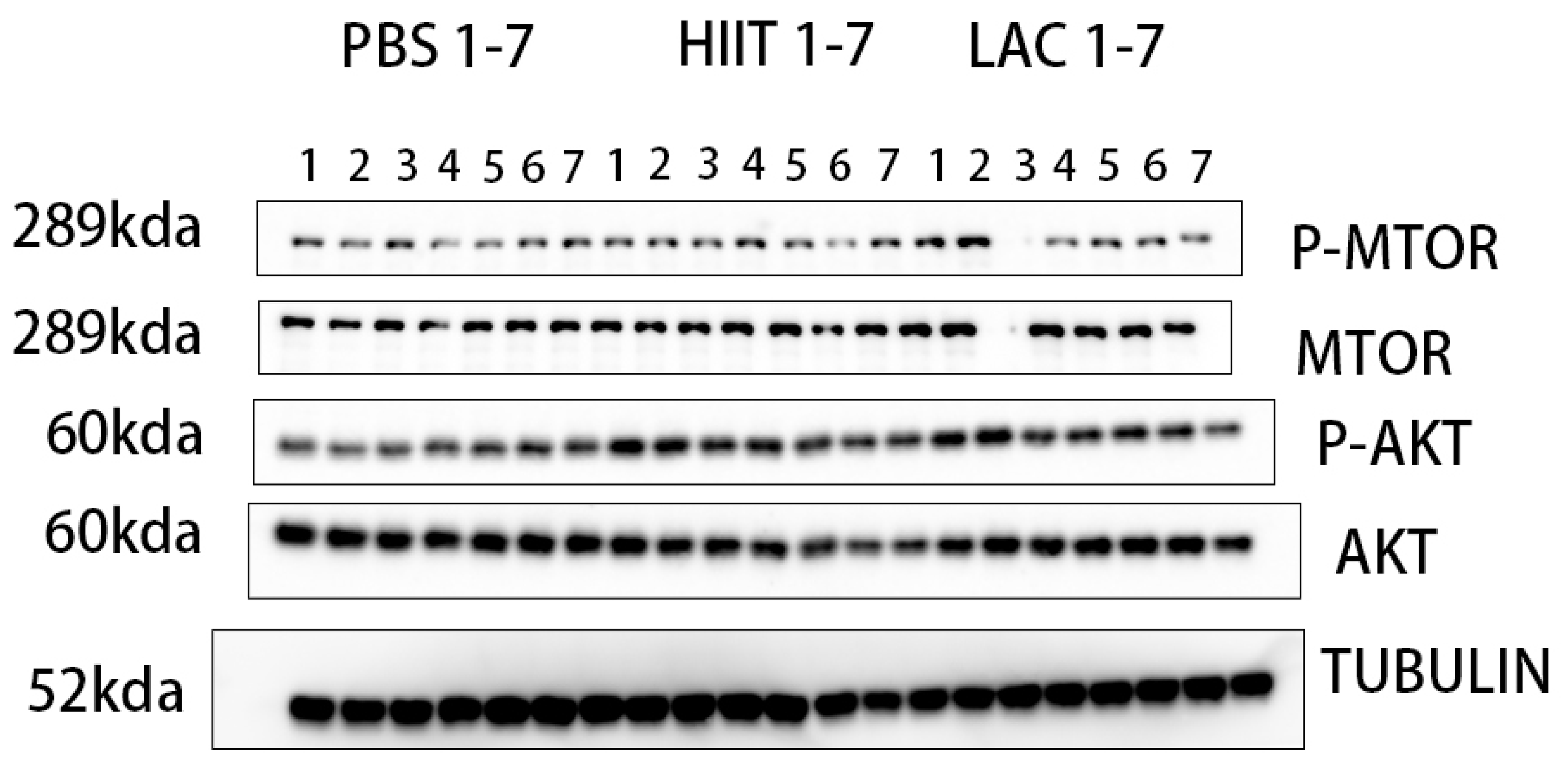

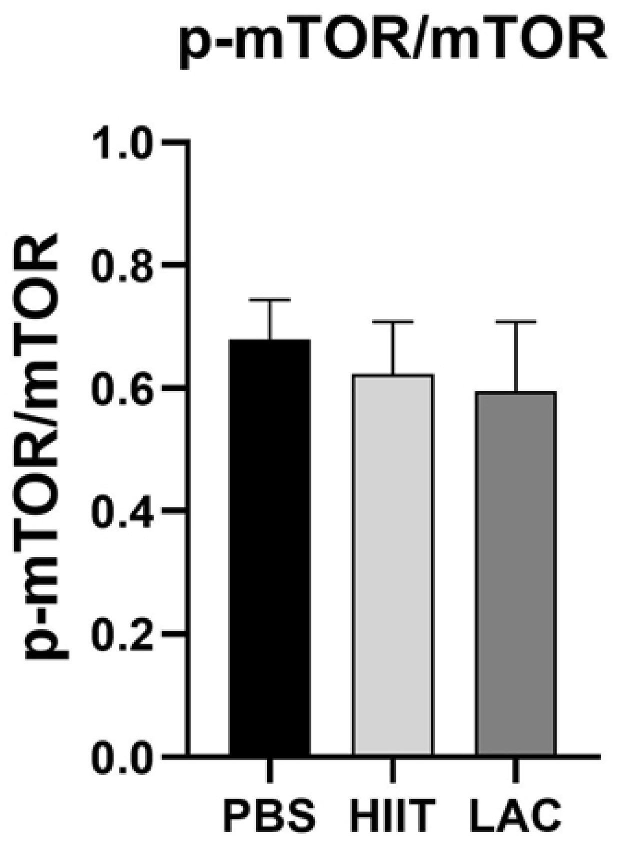

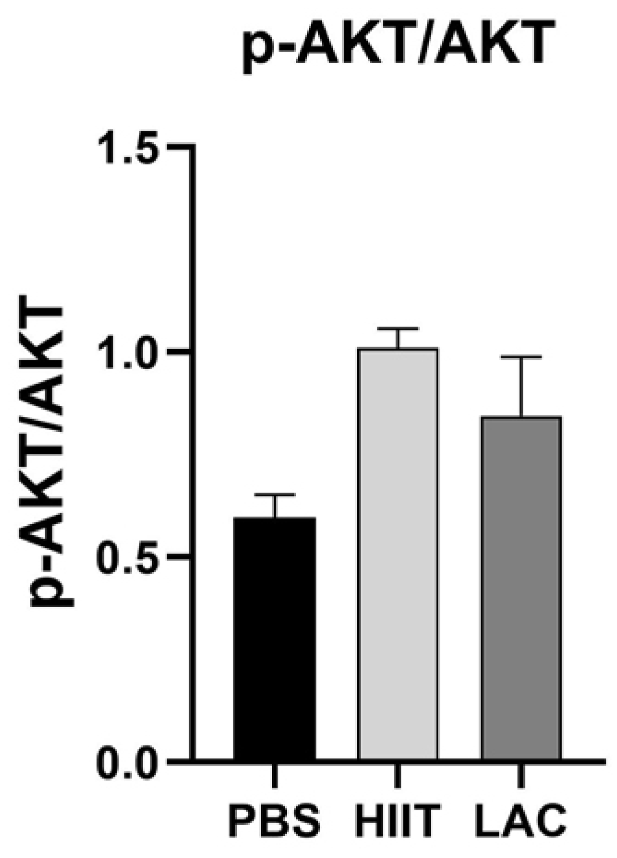

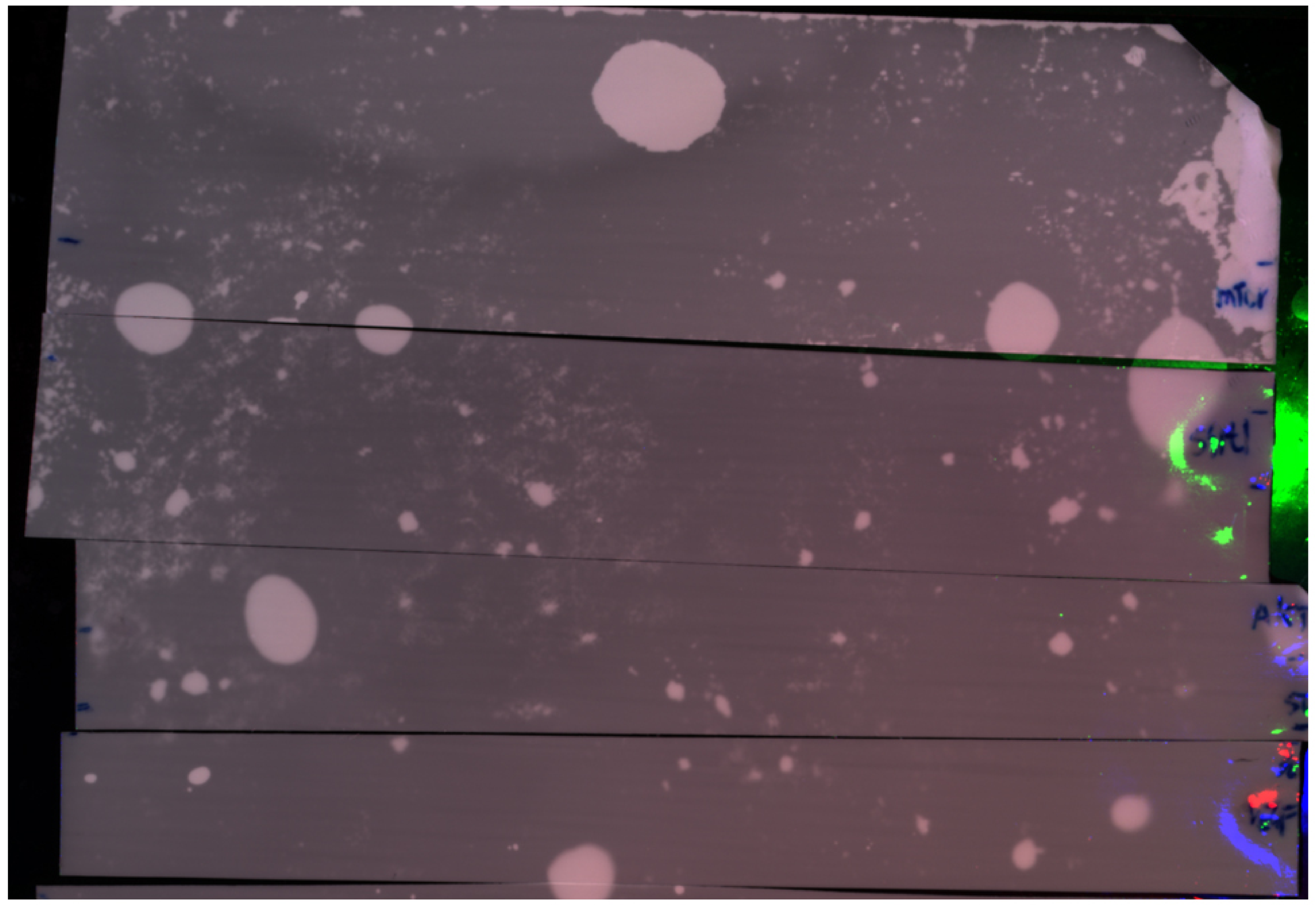

DAY1 P-MTOR DAY2 MTOR

DAY1 P-AKT DAY2 AKT DAY3 TUBULIN

DAY1

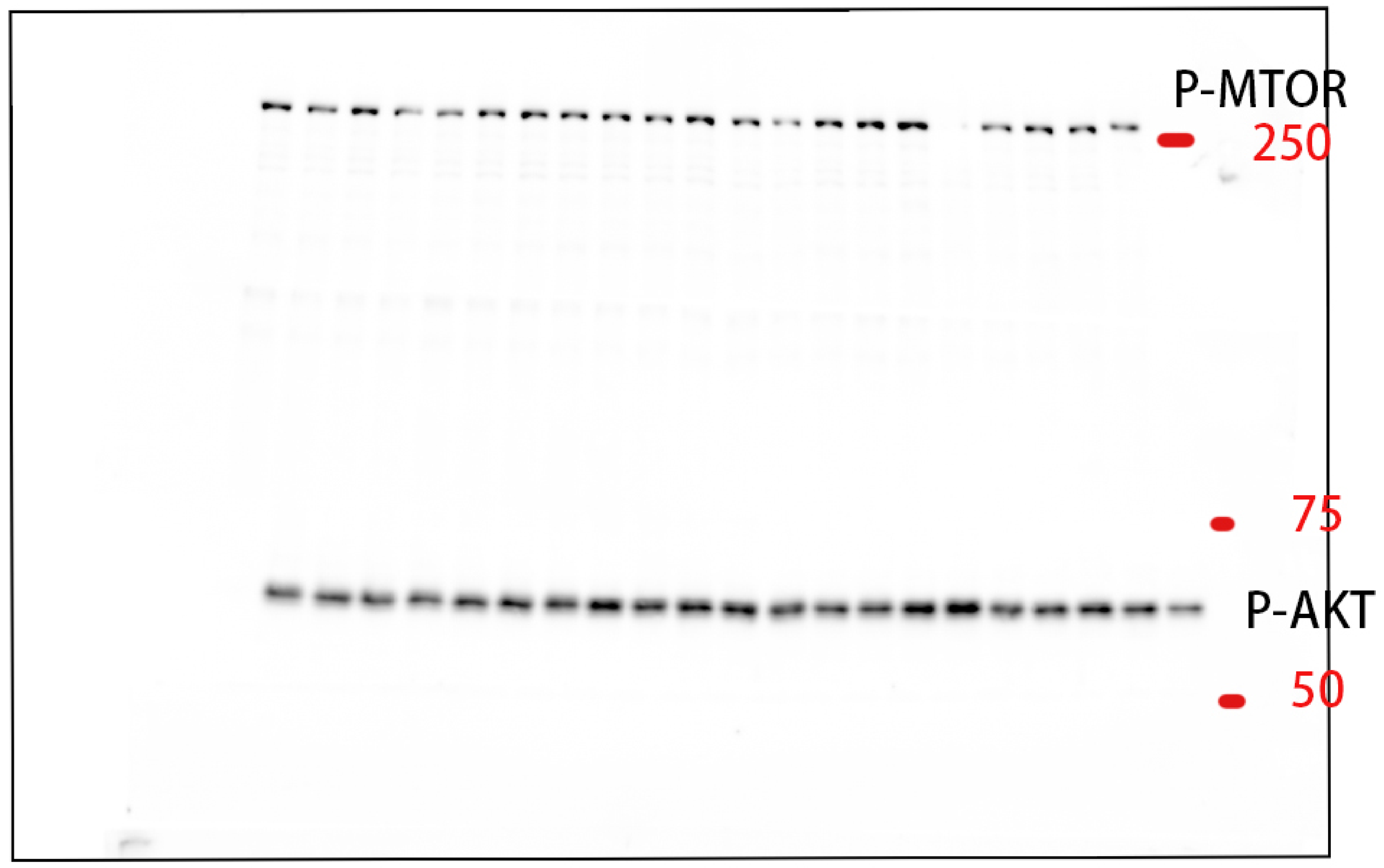

DAY2

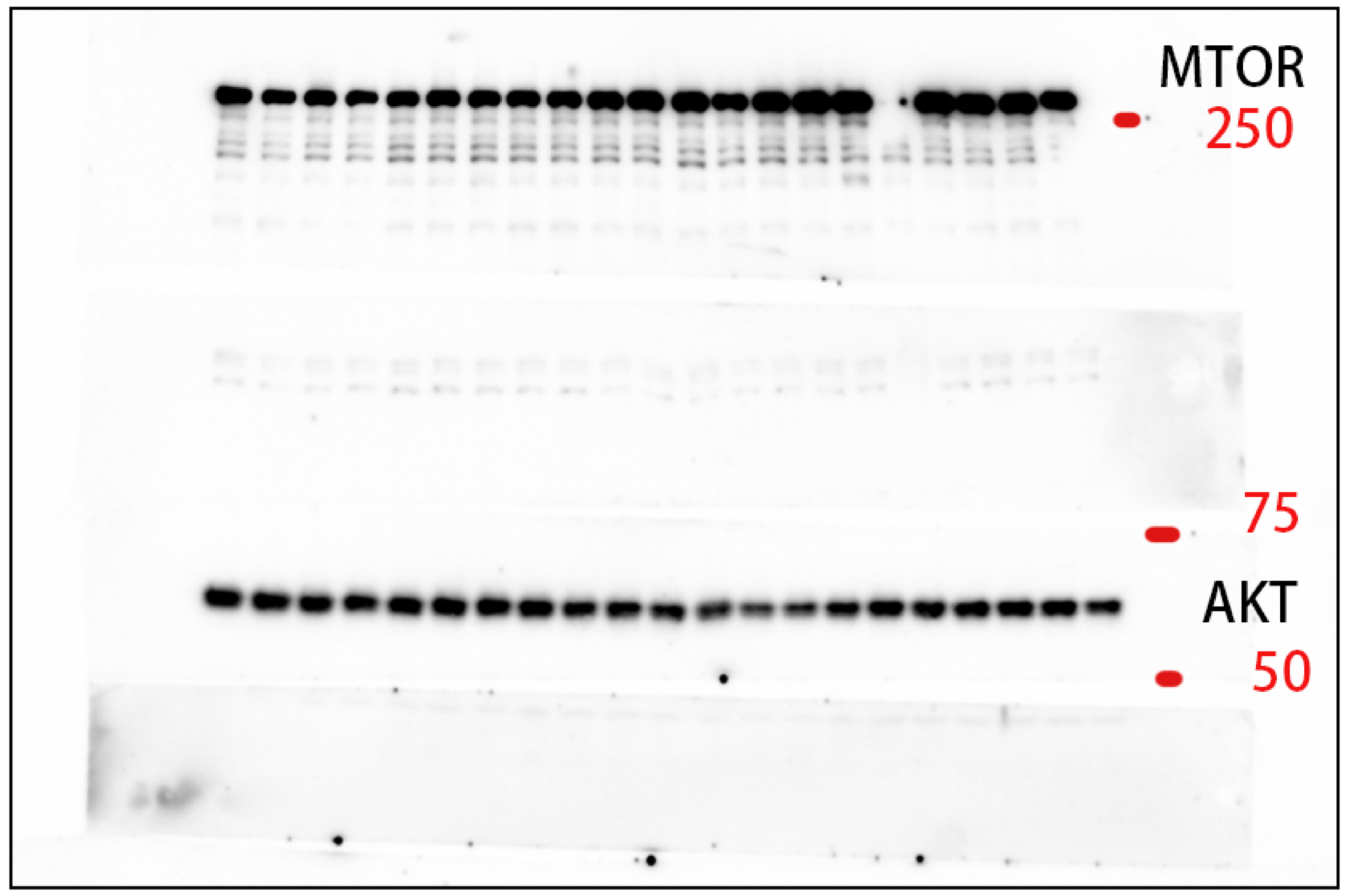

DAY3

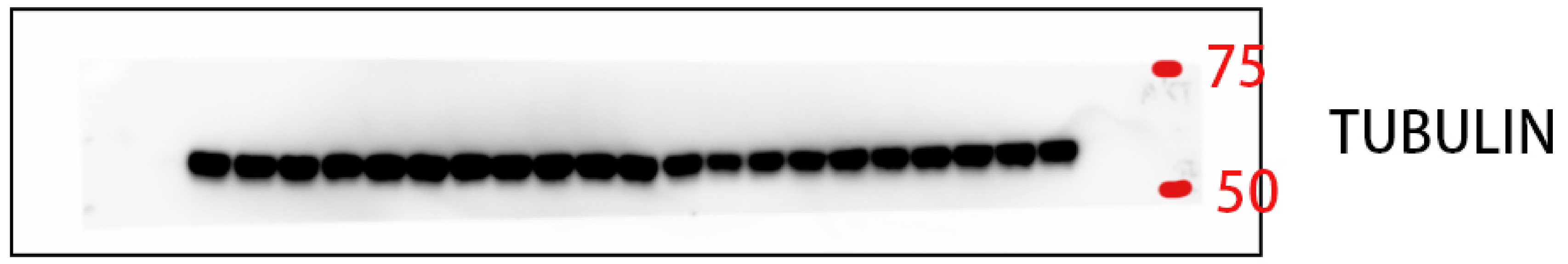

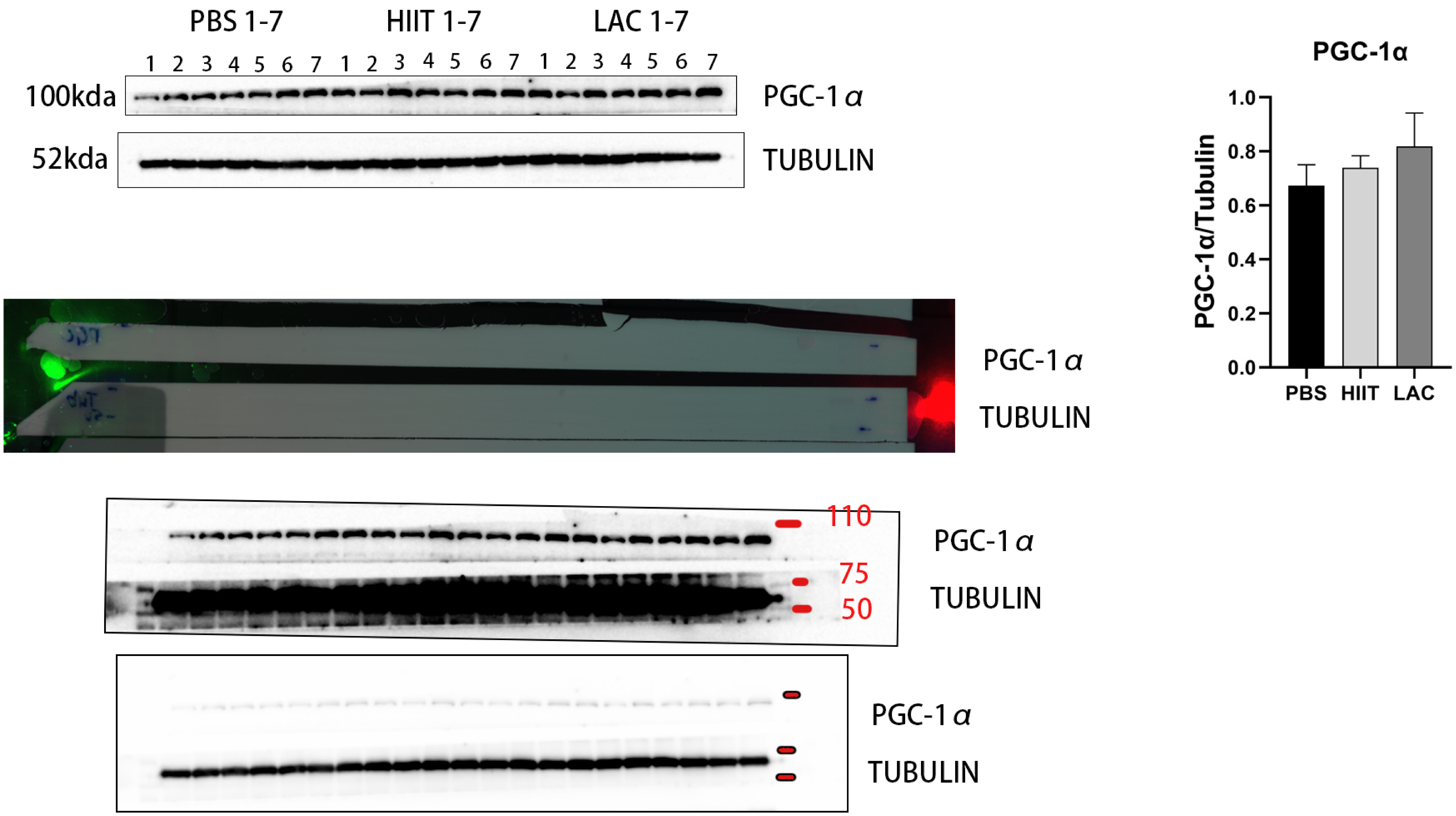
PBS 1-7
HIIT 1-7
LAC 1-7
1 2 3 4 5 6 7 1 2 3 4 5 6 7 1 2 3 4 5 6 7
100kda
PGC-1α
52kda
TUBULIN
PGC-1α
TUBULIN
110
75
50
PGC-1α
TUBULIN
PGC-1α
TUBULIN
PGC-1α
PGC-1α/Tubulin
1.0
0.8
0.6
0.4
0.2
0.0
PBS
HIIT
LAC

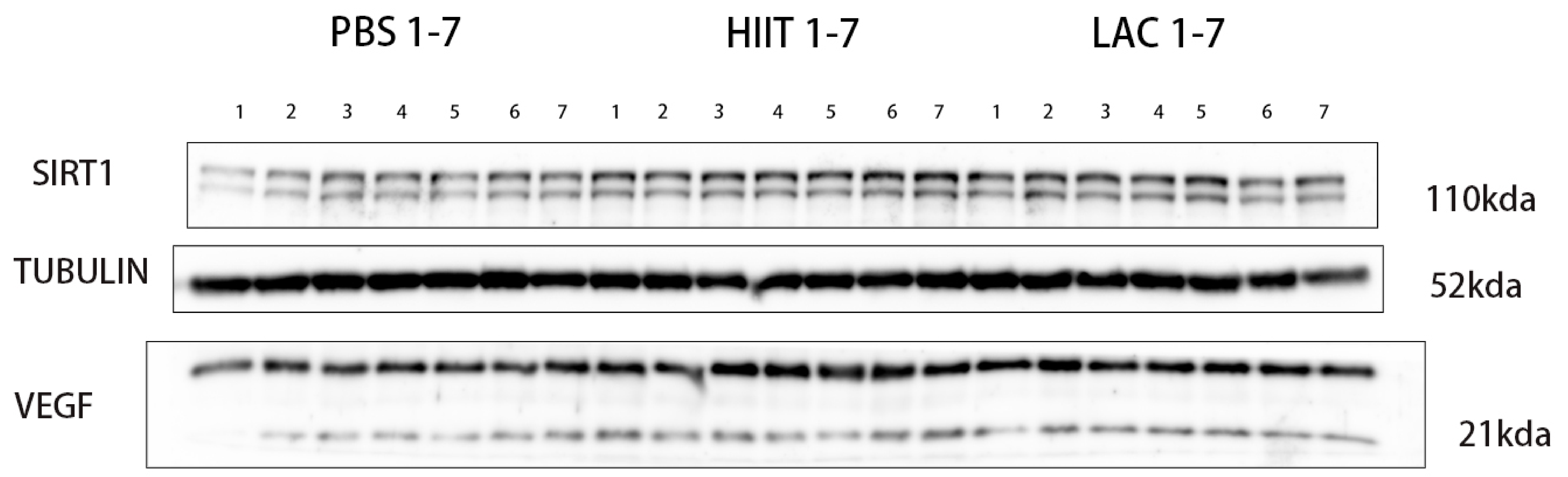
PBS 1-7
HIIT 1-7
LAC 1-7
1 2 3 4 5 6 7 1 2 3 4 5 6 7 1 2 3 4 5 6 7
SIRT1
TUBULIN
VEGF
110kda
52kda
21kda

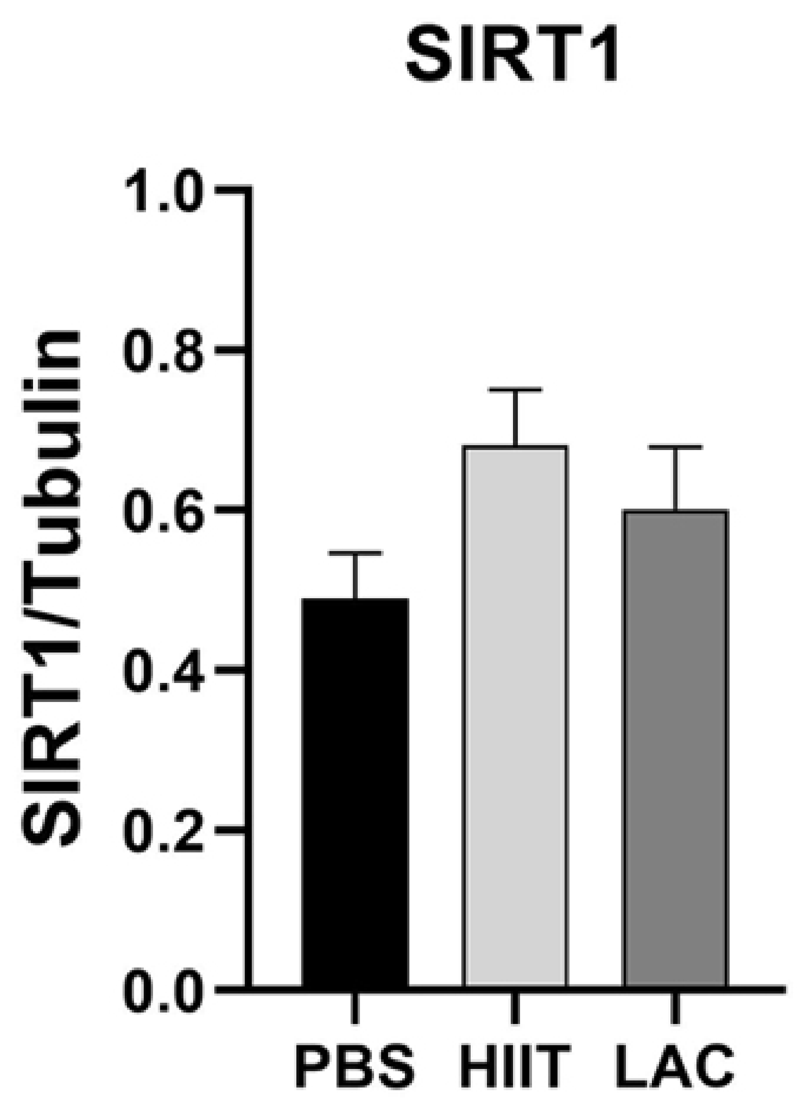
SIRT1
SIRT1/Tubulin
1.0
0.8
0.6
0.4
0.2
0.0
PBS
HIIT
LAC

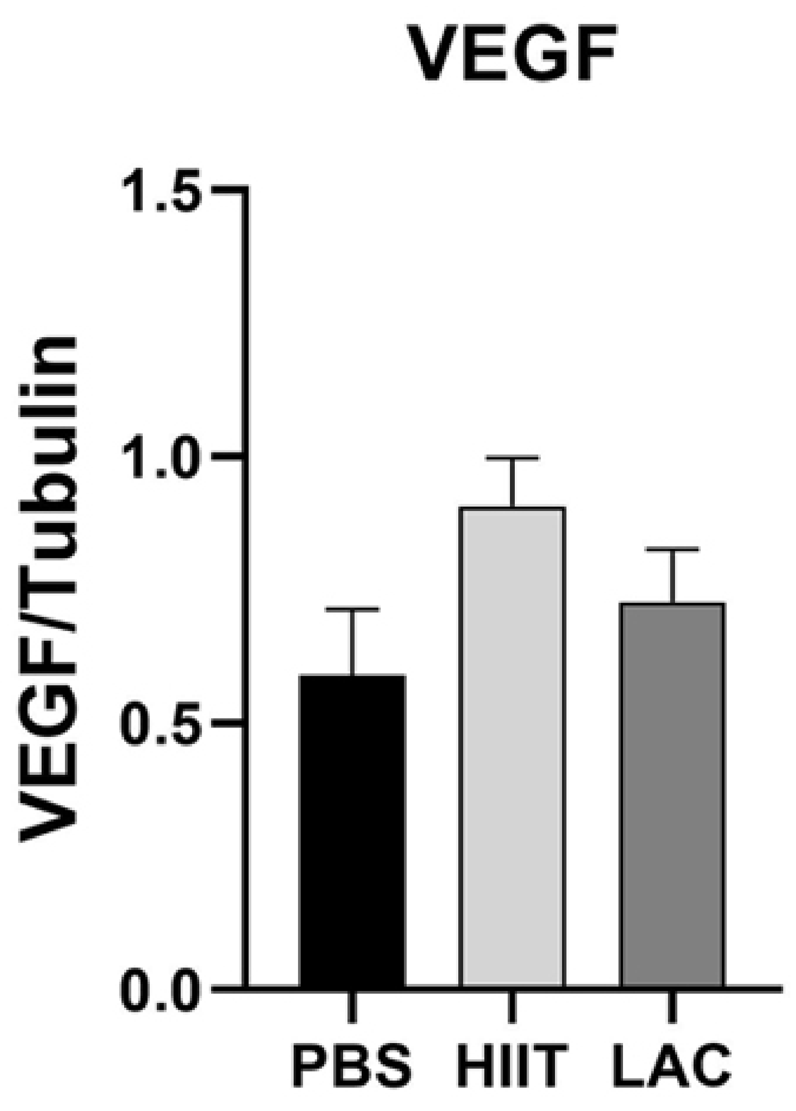
VEGF
VEGF/Tubulin
1.5
1.0
0.5
0.0
PBS
HIIT
LAC

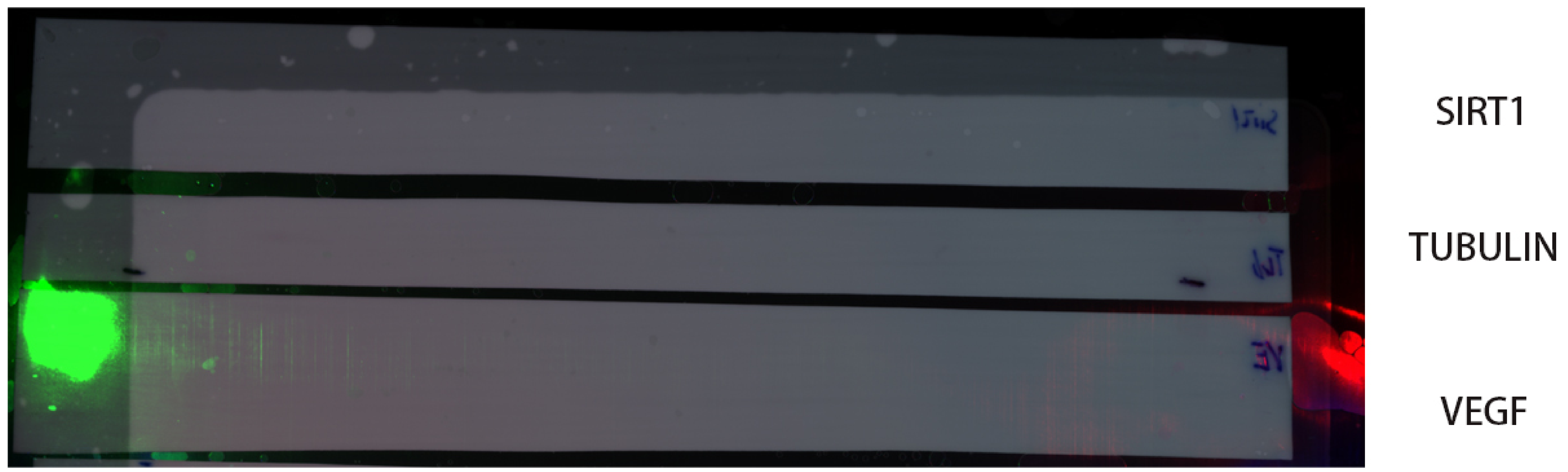
SIRT1
TUBULIN
VEGF

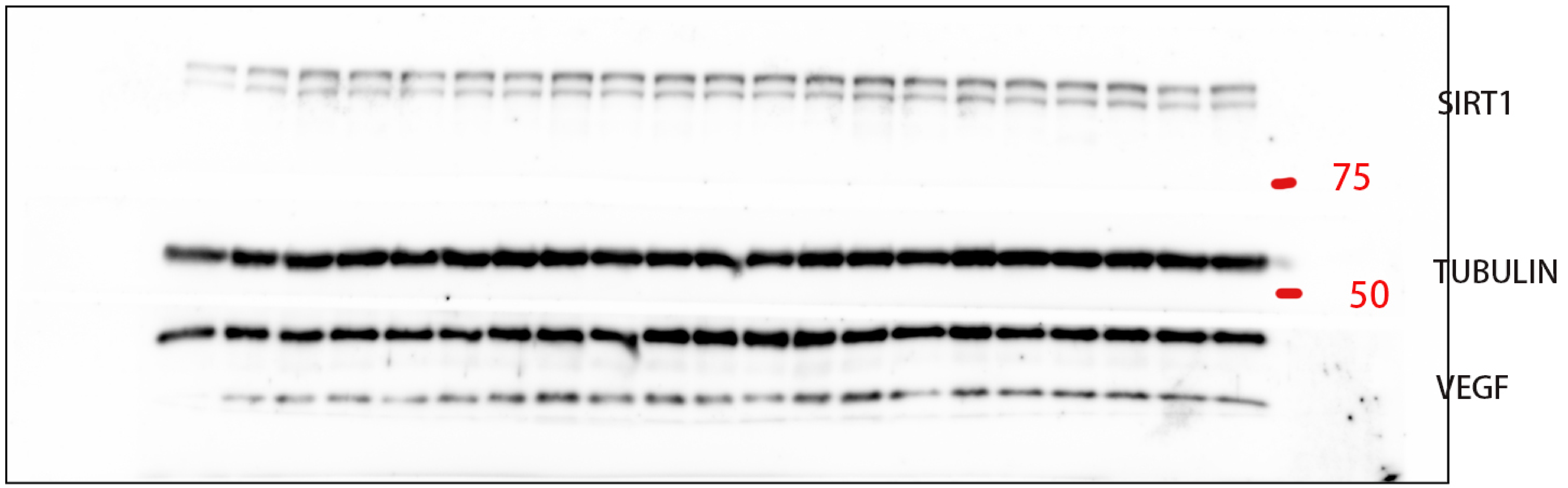
SIRT1
75
TUBULIN
50
VEGF

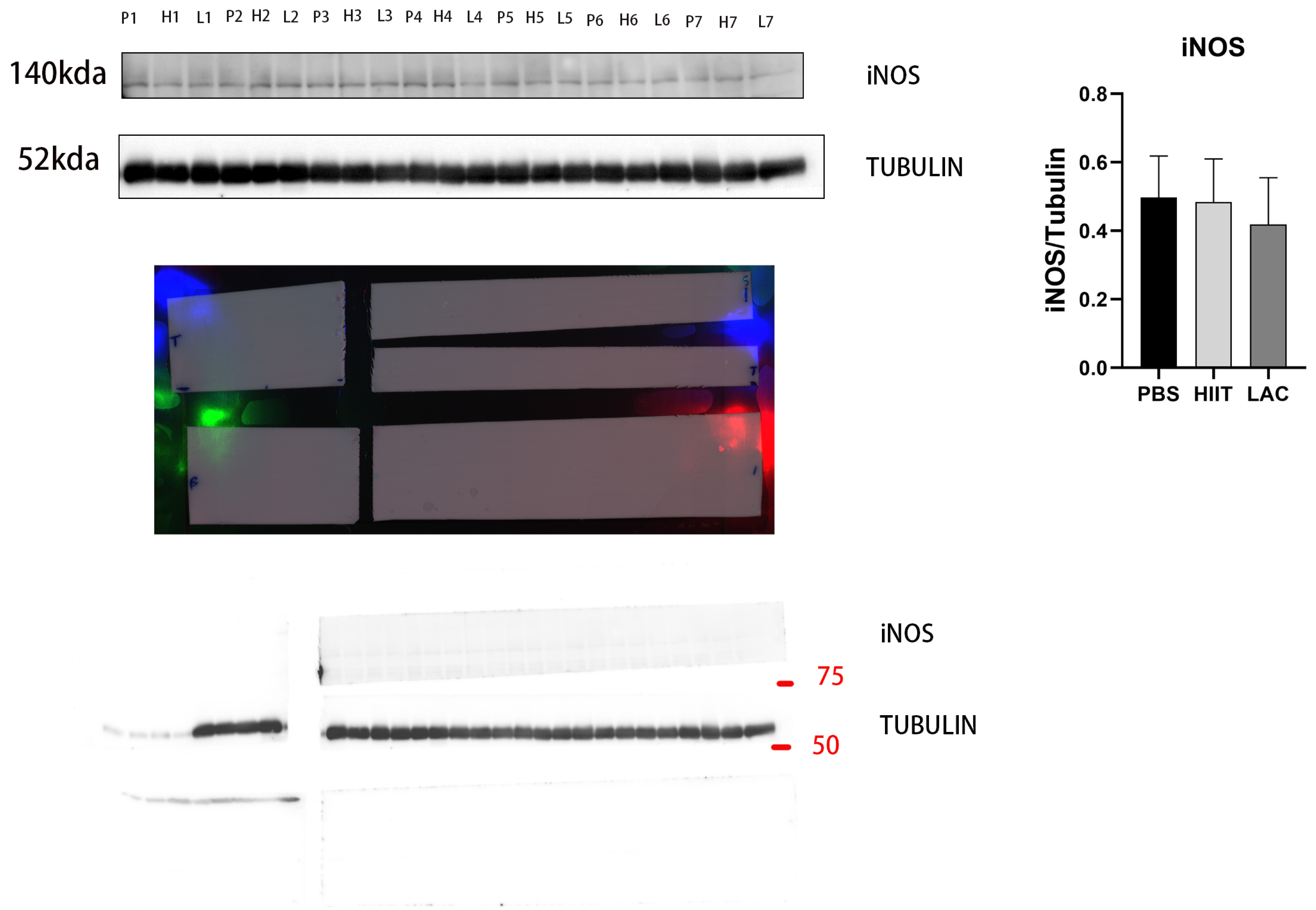
P1 H1 L1 P2 H2 L2 P3 H3 L3 P4 H4 L4 P5 H5 L5 P6 H6 L6 P7 H7 L7
140kda
iNOS
52kda
TUBULIN
iNOS
iNOS/Tubulin
0.0
0.2
0.4
0.6
0.8
PBS
HIIT
LAC
iNOS
75
TUBULIN
50

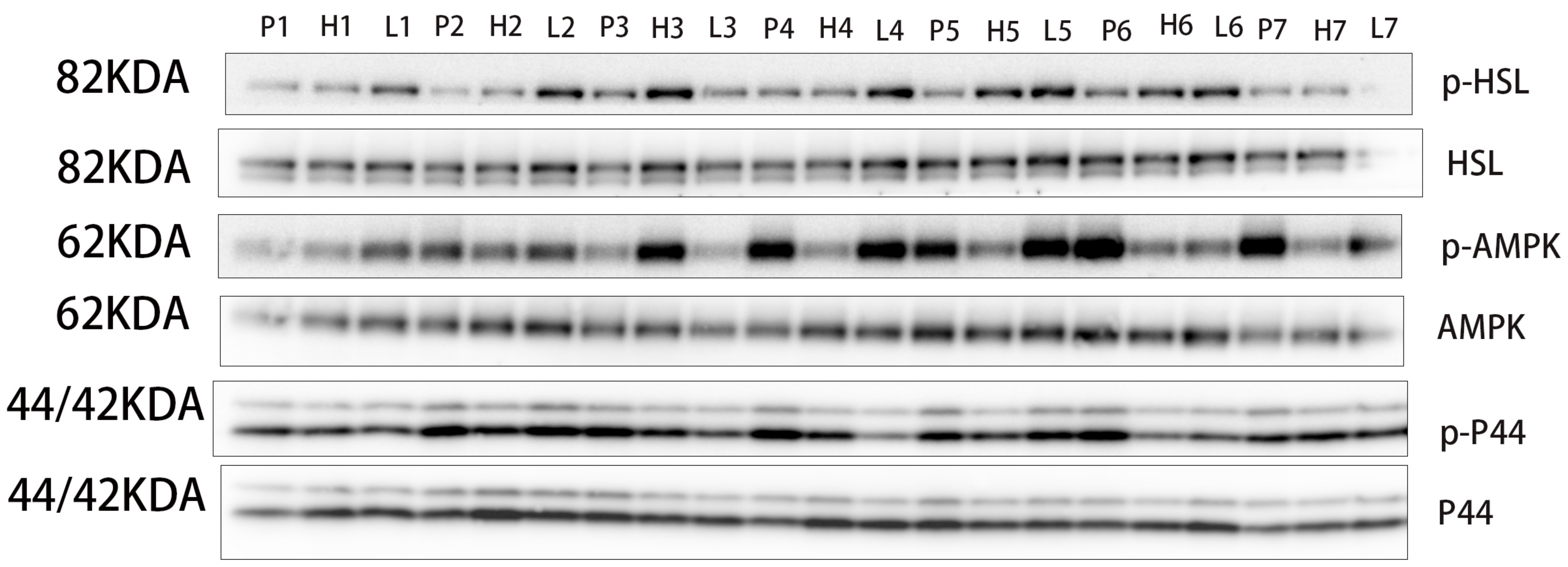
P1 H1 L1 P2 H2 L2 P3 H3 L3 P4 H4 L4 P5 H5 L5 P6 H6 L6 P7 H7 L7
82KDA
p-HSL
82KDA
HSL
62KDA
p-AMPK
62KDA
AMPK
44/42KDA
p-P44
44/42KDA
P44

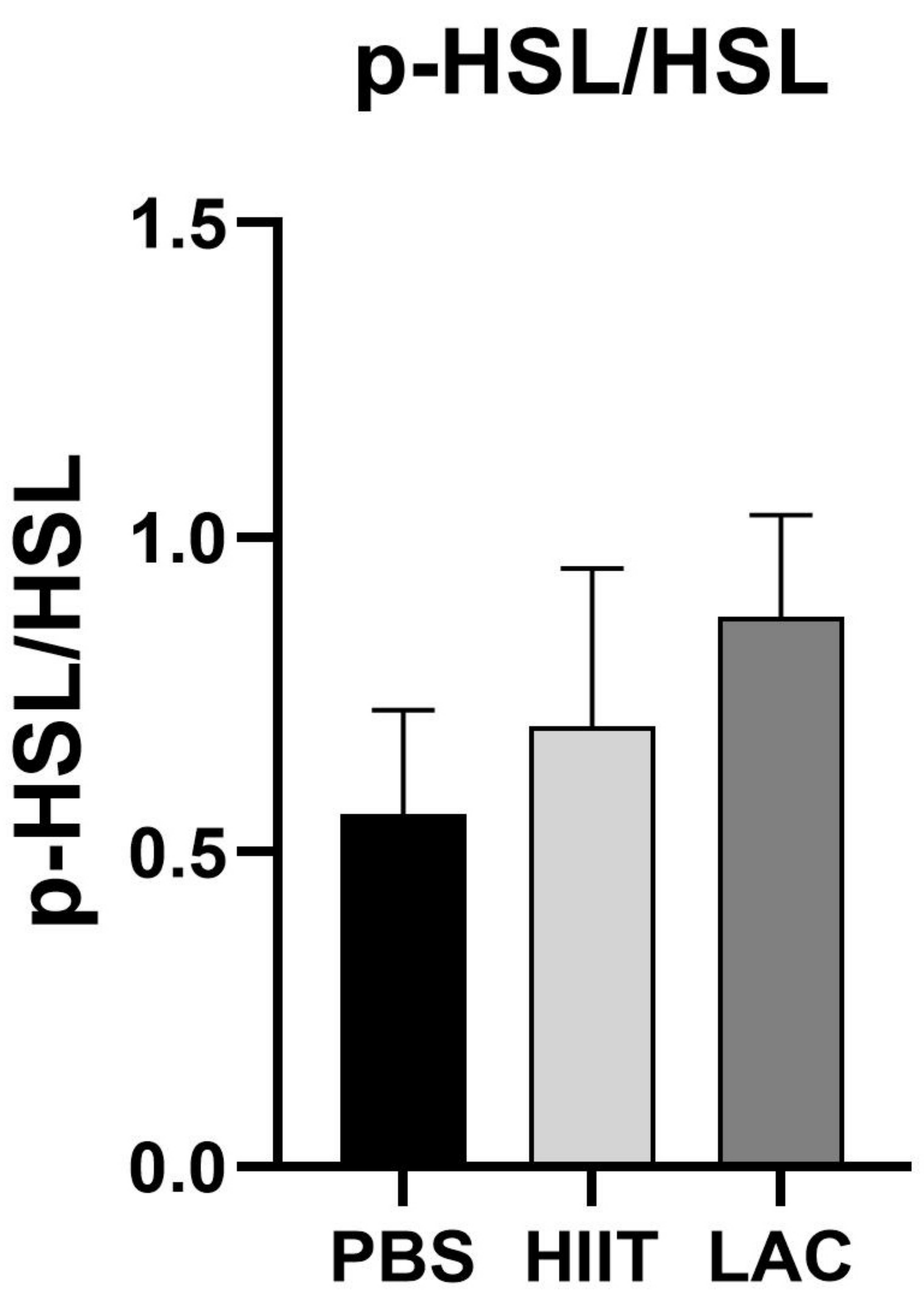
p-HSL/HSL
p-HSL/HSL
1.5
1.0
0.5
0.0
PBS
HIIT
LAC

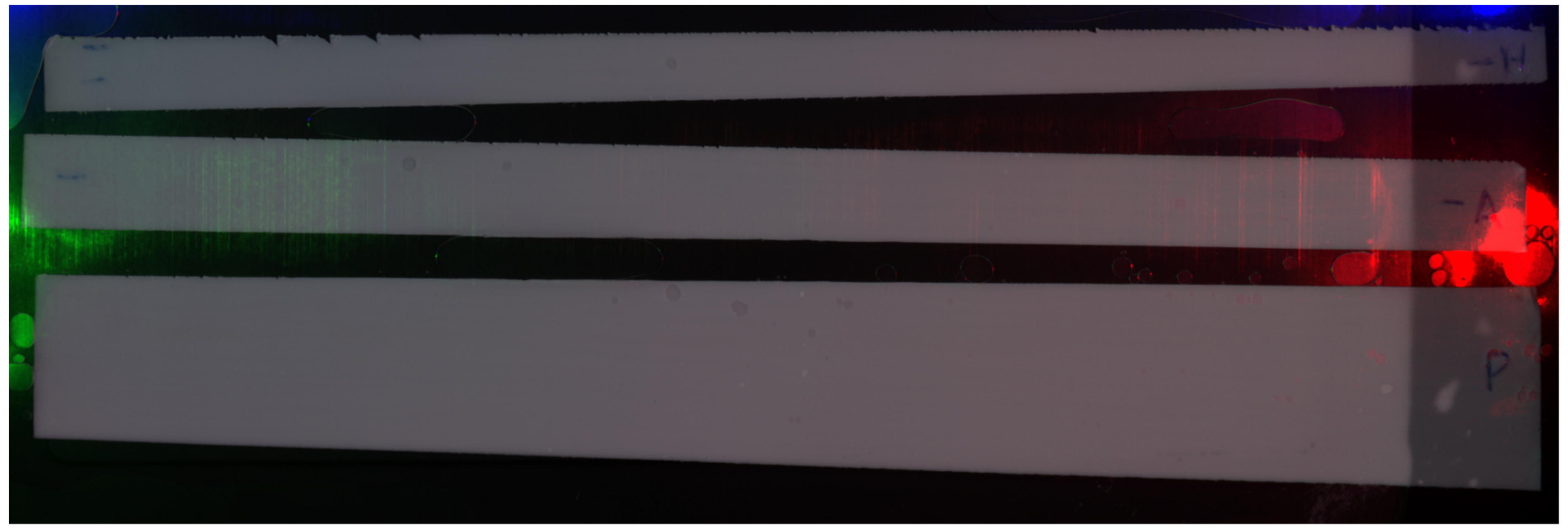

DAY1

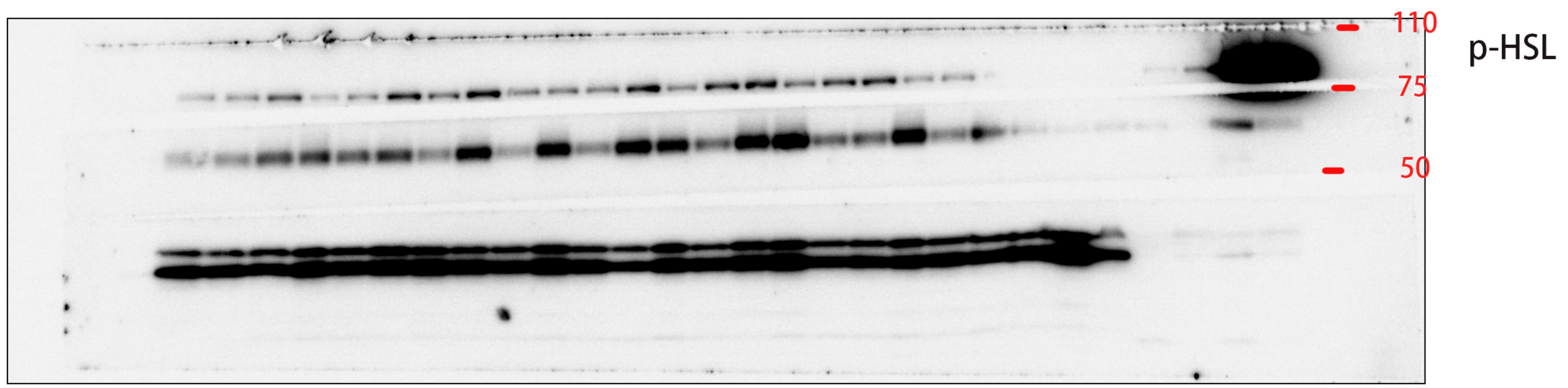
p-HSL
110
75
50

DAY2

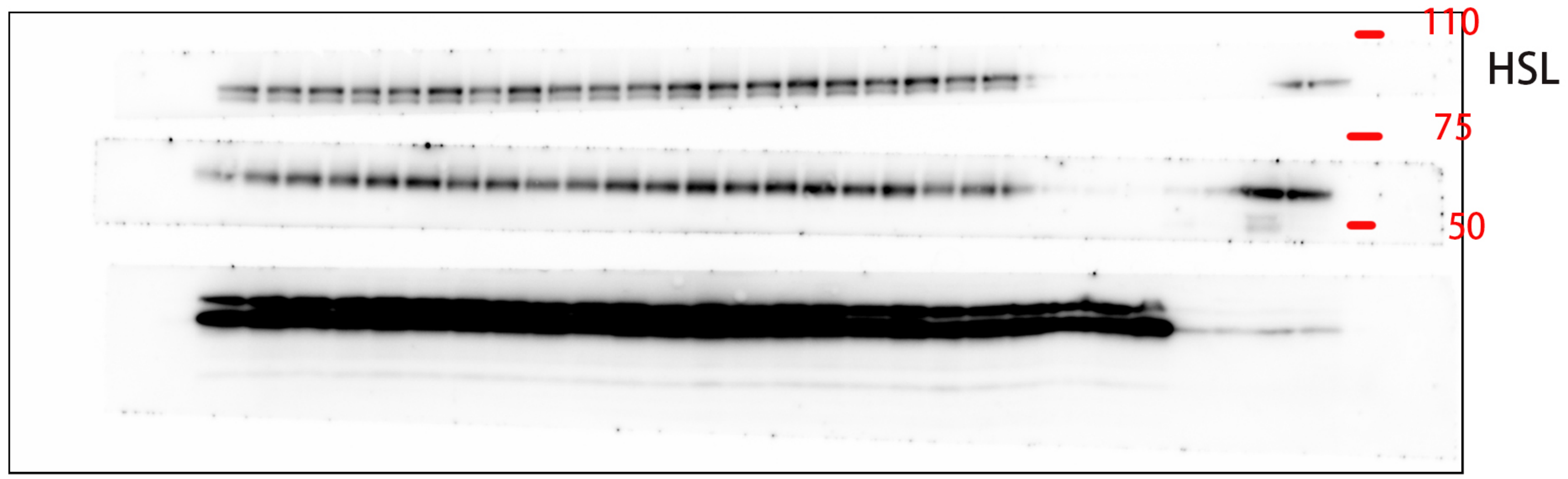
HSL
110
75
50

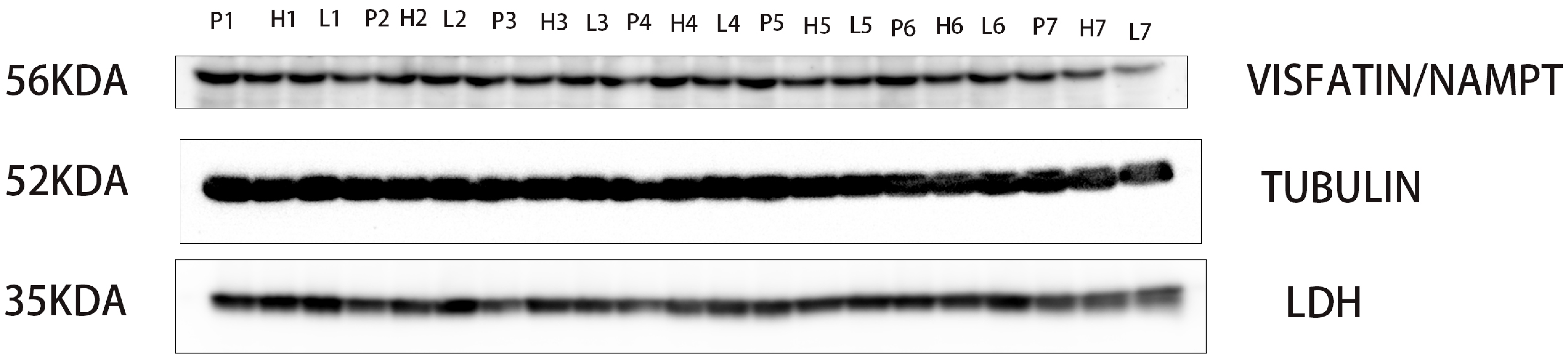
P1 H1 L1 P2 H2 L2 P3 H3 L3 P4 H4 L4 P5 H5 L5 P6 H6 L6 P7 H7 L7
56KDA
VISFATIN/NAMPT
52KDA
TUBULIN
35KDA
LDH

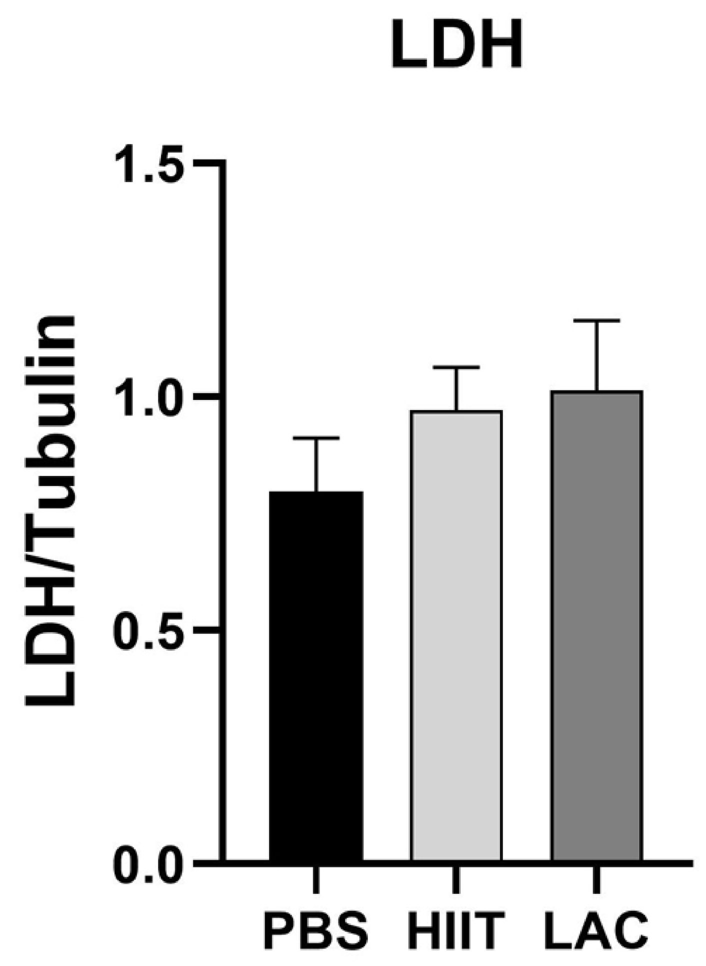
LDH
LDH/Tubulin
1.5
1.0
0.5
0.0
PBS
HIIT
LAC

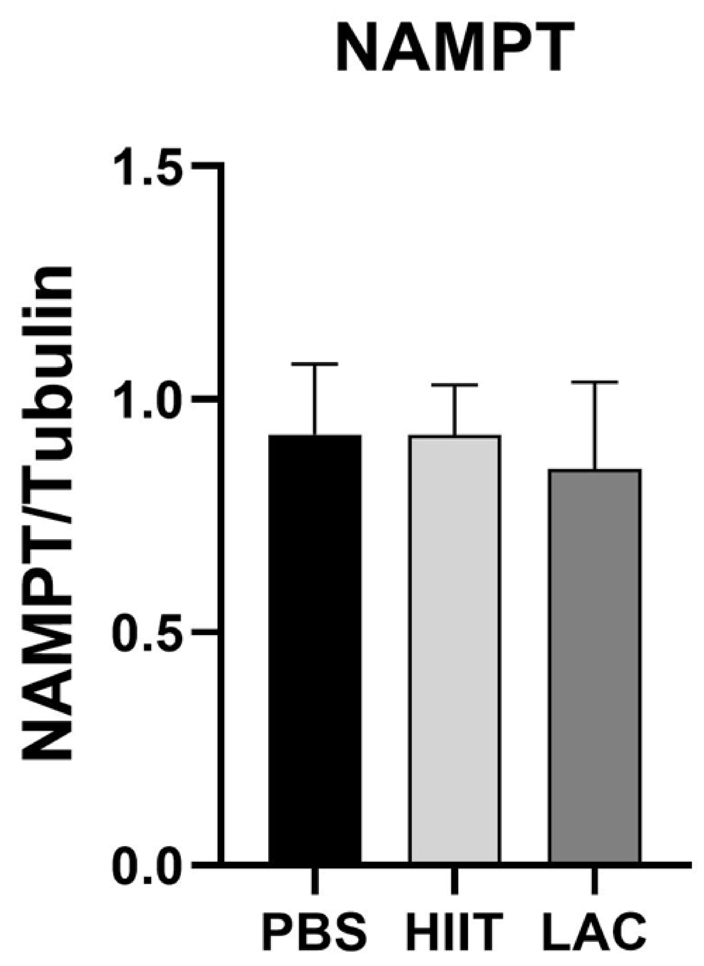
NAMPT
NAMPT/Tubulin
1.5
1.0
0.5
0.0
PBS
HIIT
LAC

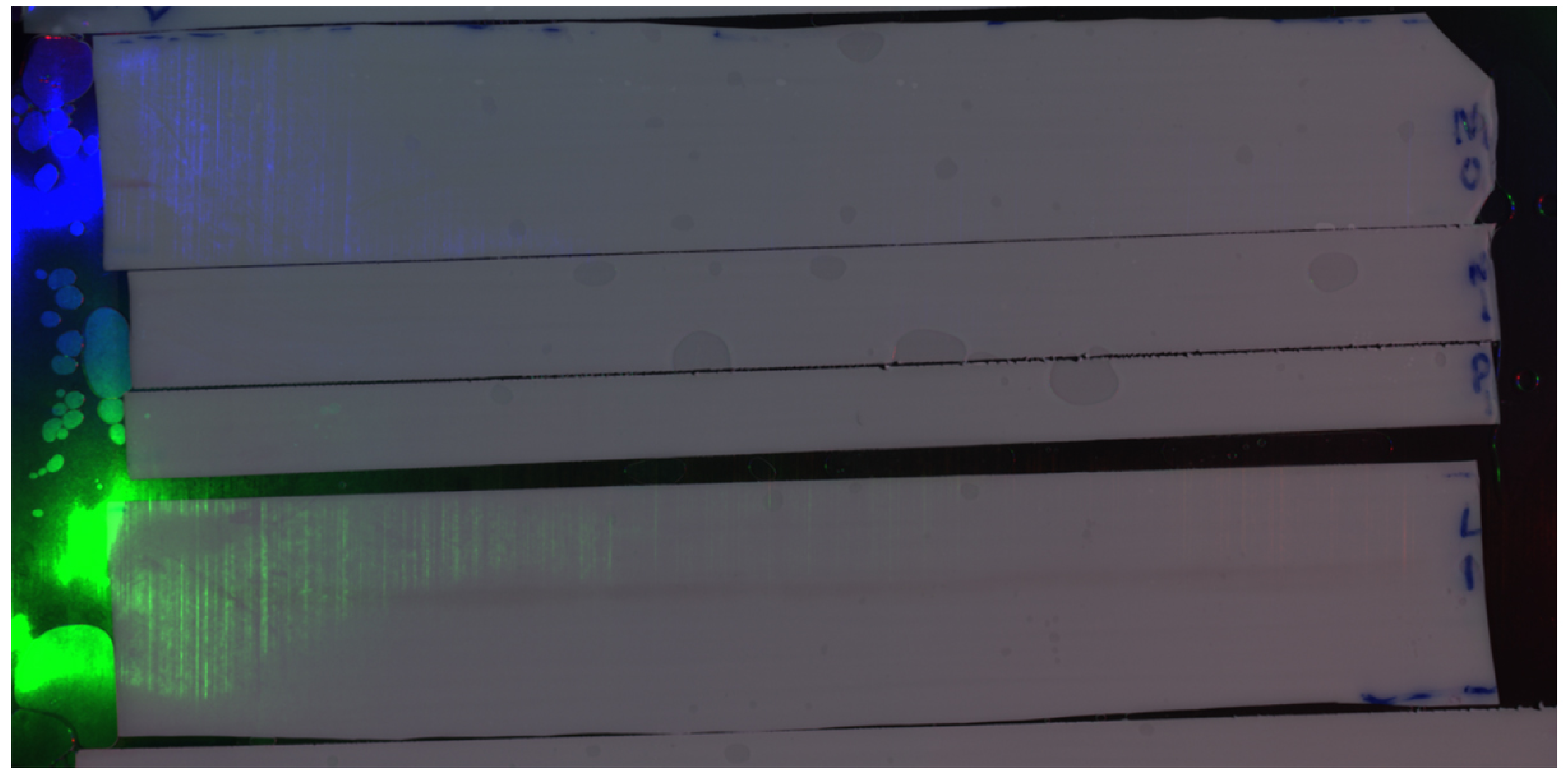

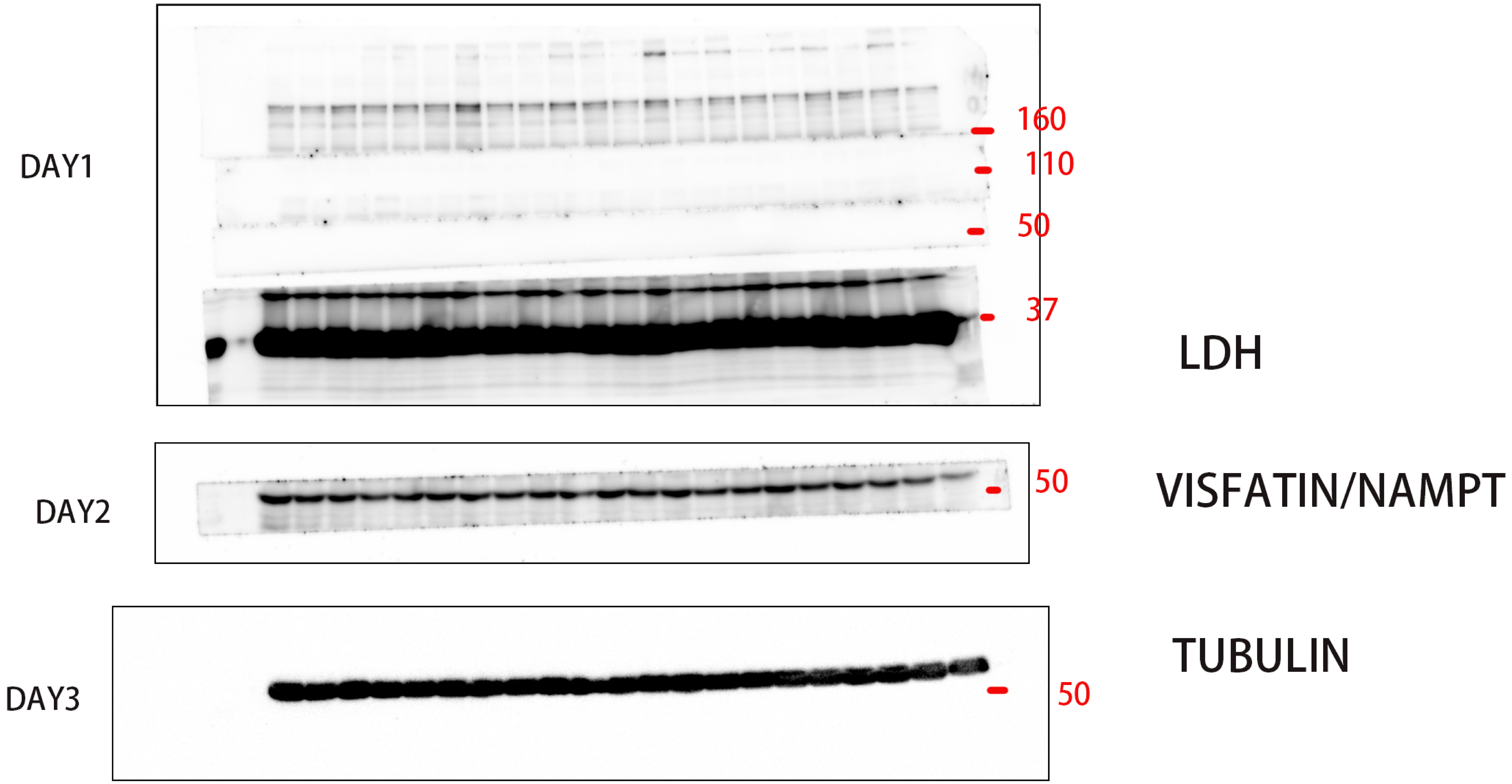
DAY1
160
110
50
37
LDH
DAY2
50
VISFATIN/NAMPT
DAY3
50
TUBULIN

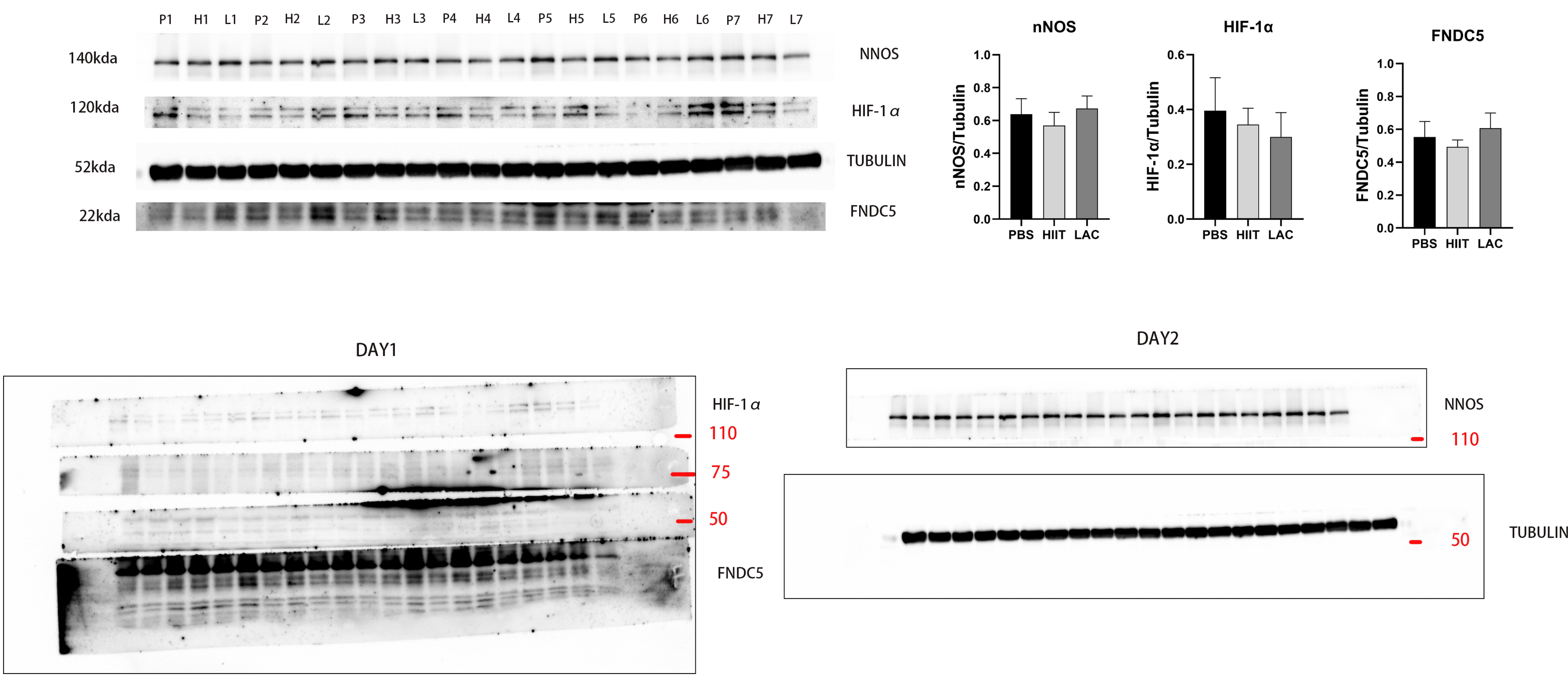

P1 H1 L1 P2 H2 L2 P3 H3 L3 P4 H4 L4 P5 H5 L5 P6 H6 L6 P7 H7 L7
140kda
NNOS
120kda
HIF-1 α
52kda
TUBULIN
22kda
FNDC5
nNOS
nNOS/Tubulin
1.0
0.8
0.6
0.4
0.2
0.0
PBS HIIT LAC
HIF-1α
HIF-1α/Tubulin
0.6
0.4
0.2
0.0
PBS HIIT LAC
FNDC5
FNDC5/Tubulin
1.0
0.8
0.6
0.4
0.2
0.0
PBS HIIT LAC
DAY1
HIF-1 α
110
75
50
FNDC5
DAY2
NNOS
110
TUBULIN
50

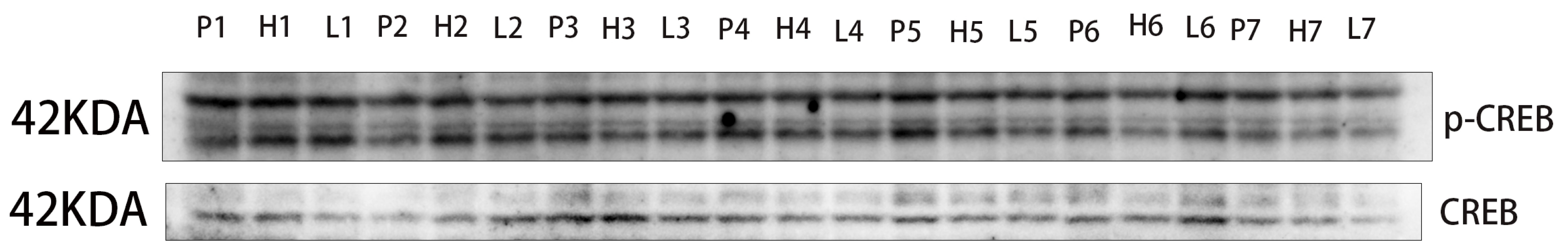
P1 H1 L1 P2 H2 L2 P3 H3 L3 P4 H4 L4 P5 H5 L5 P6 H6 L6 P7 H7 L7
42KDA
p-CREB
42KDA
CREB

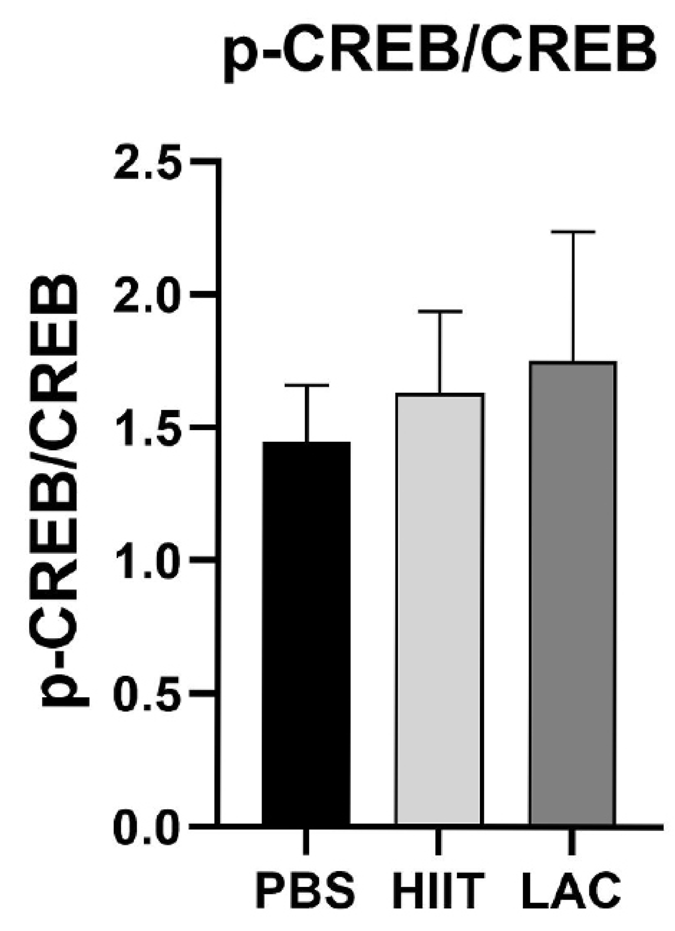
p-CREB/CREB
p-CREB/CREB
2.5
2.0
1.5
1.0
0.5
0.0
PBS
HIIT
LAC

DAY1

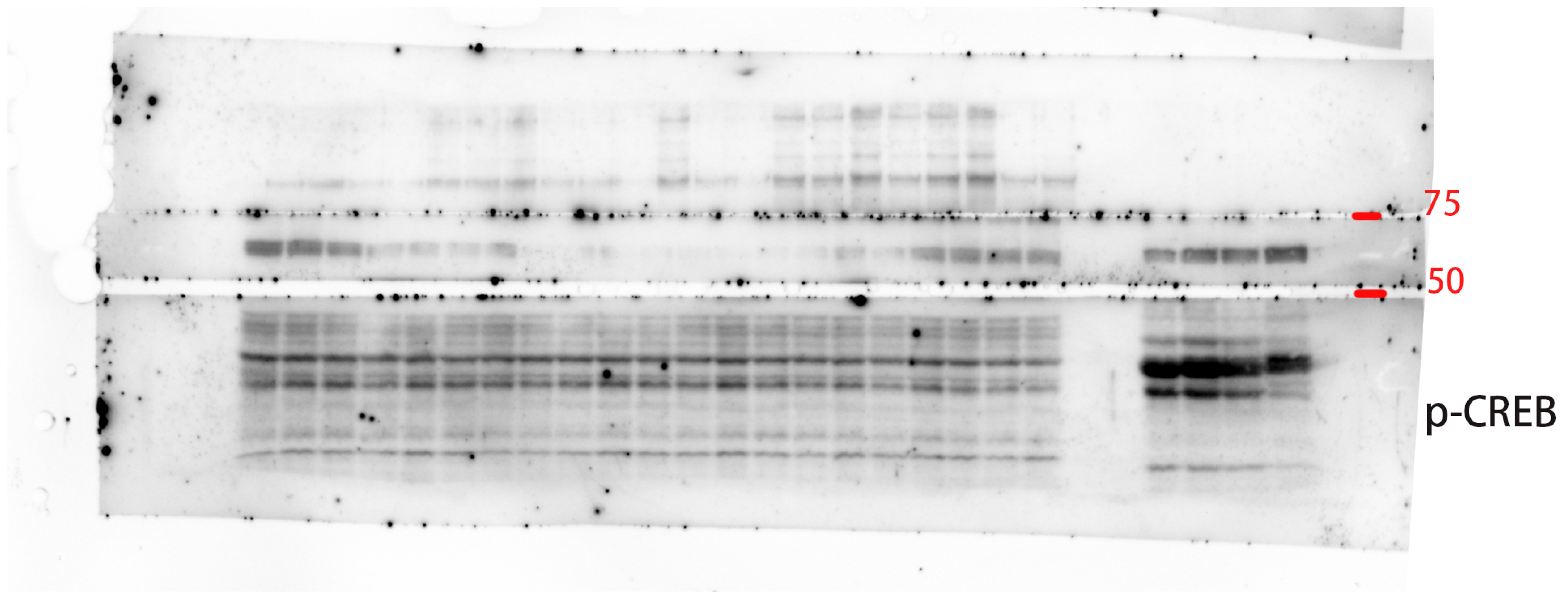
75
50
p-CREB

DAY2

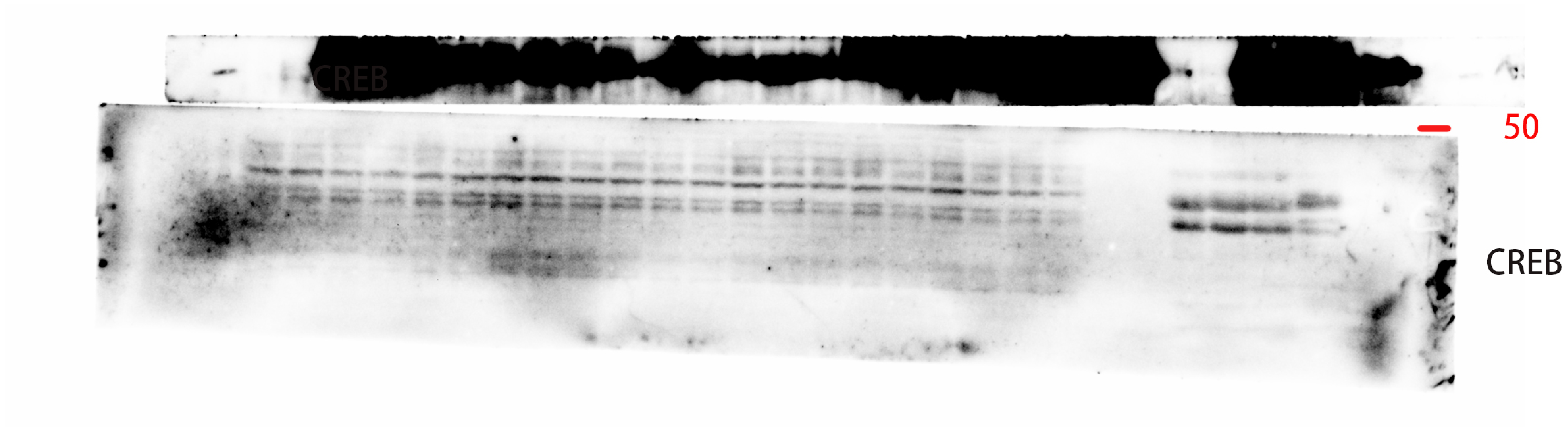
50
CREB

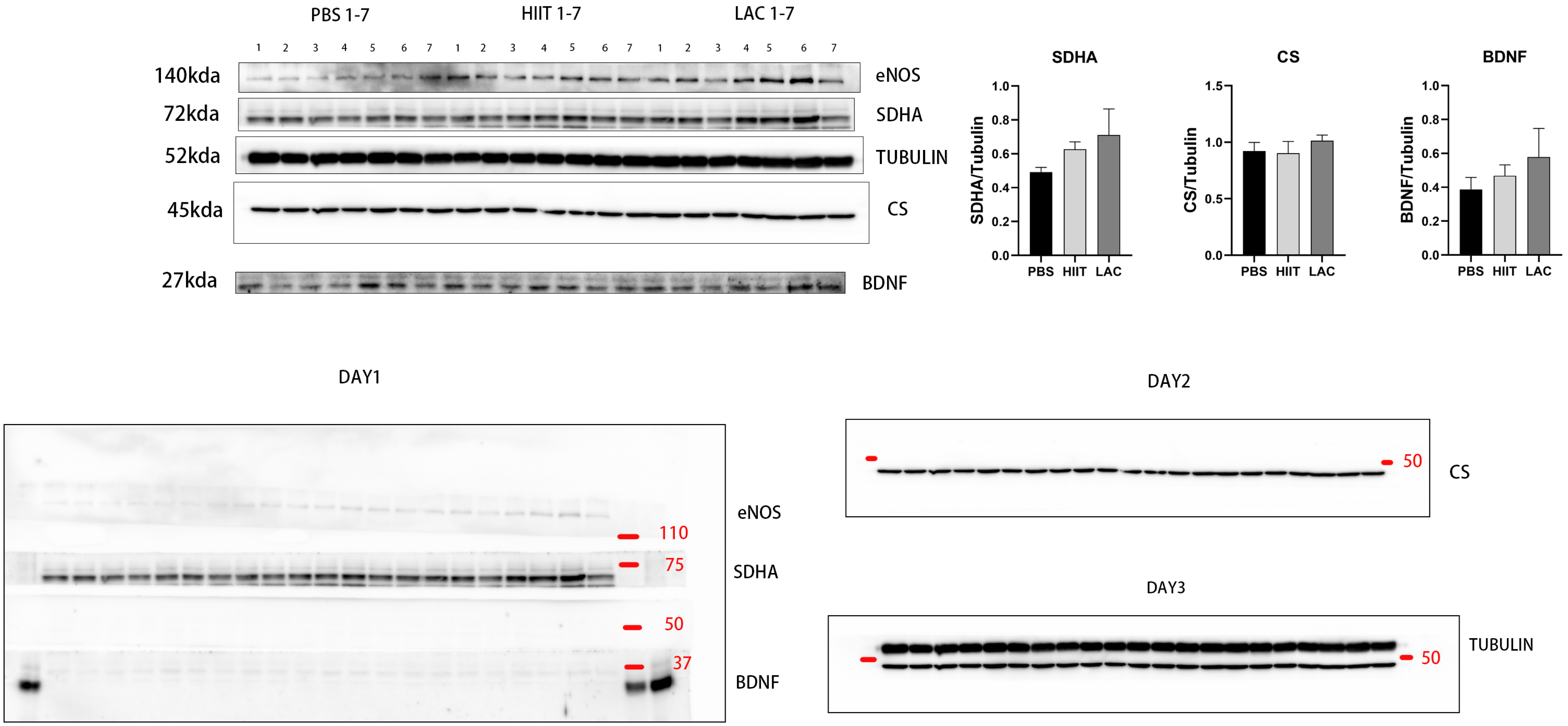
PBS 1-7
HIIT 1-7
LAC 1-7
1 2 3 4 5 6 7 1 2 3 4 5 6 7 1 2 3 4 5 6 7
140kda
eNOS
72kda
SDHA
52kda
TUBULIN
45kda
CS
27kda
BDNF
SDHA
SDHA/Tubulin
1.0
0.8
0.6
0.4
0.2
0.0
PBS HIIT LAC
CS
CS/Tubulin
1.5
1.0
0.5
0.0
PBS HIIT LAC
BDNF
BDNF/Tubulin
1.0
0.8
0.6
0.4
0.2
0.0
PBS HIIT LAC
DAY1
eNOS
110
75
SDHA
50
37
BDNF
DAY2
50
CS
DAY3
TUBULIN
50

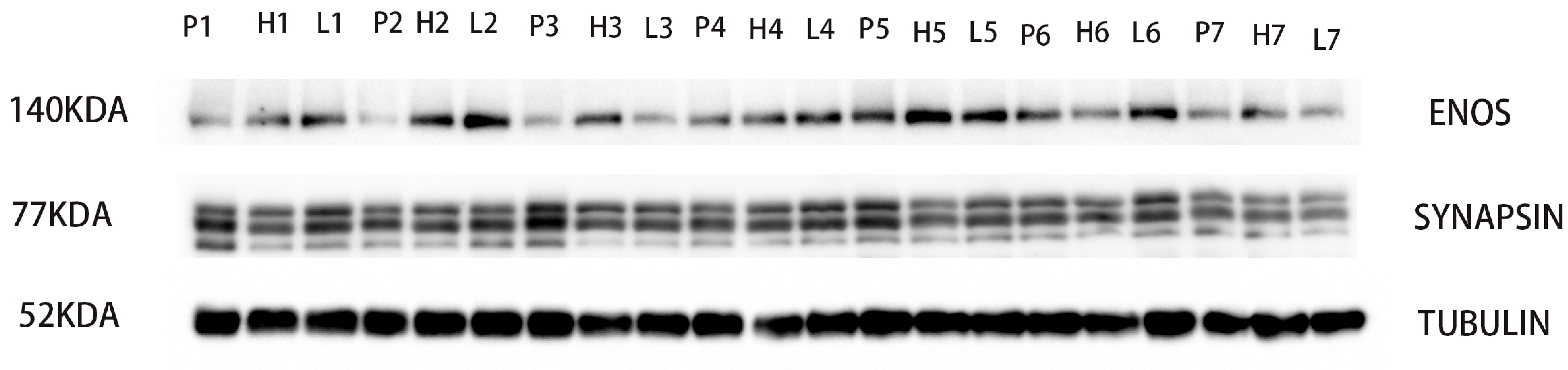
P1 H1 L1 P2 H2 L2 P3 H3 L3 P4 H4 L4 P5 H5 L5 P6 H6 L6 P7 H7 L7
140KDA
ENOS
77KDA
SYNAPSIN
52KDA
TUBULIN

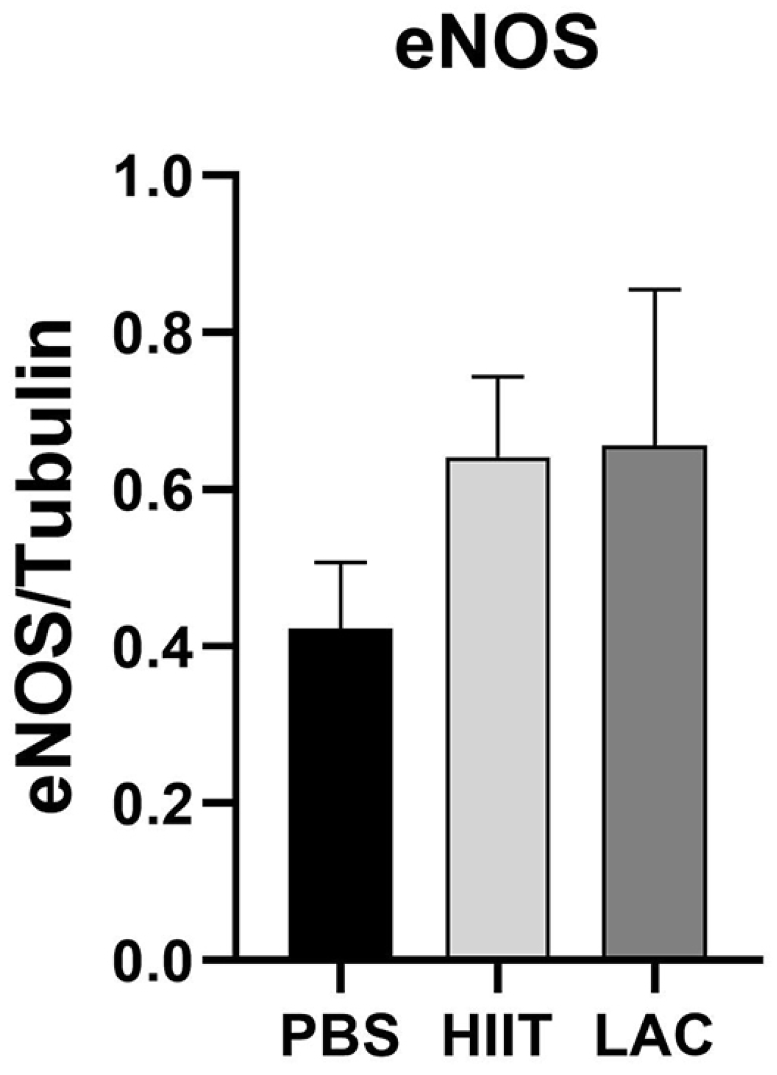
eNOS
eNOS/Tubulin
1.0
0.8
0.6
0.4
0.2
0.0
PBS
HIIT
LAC

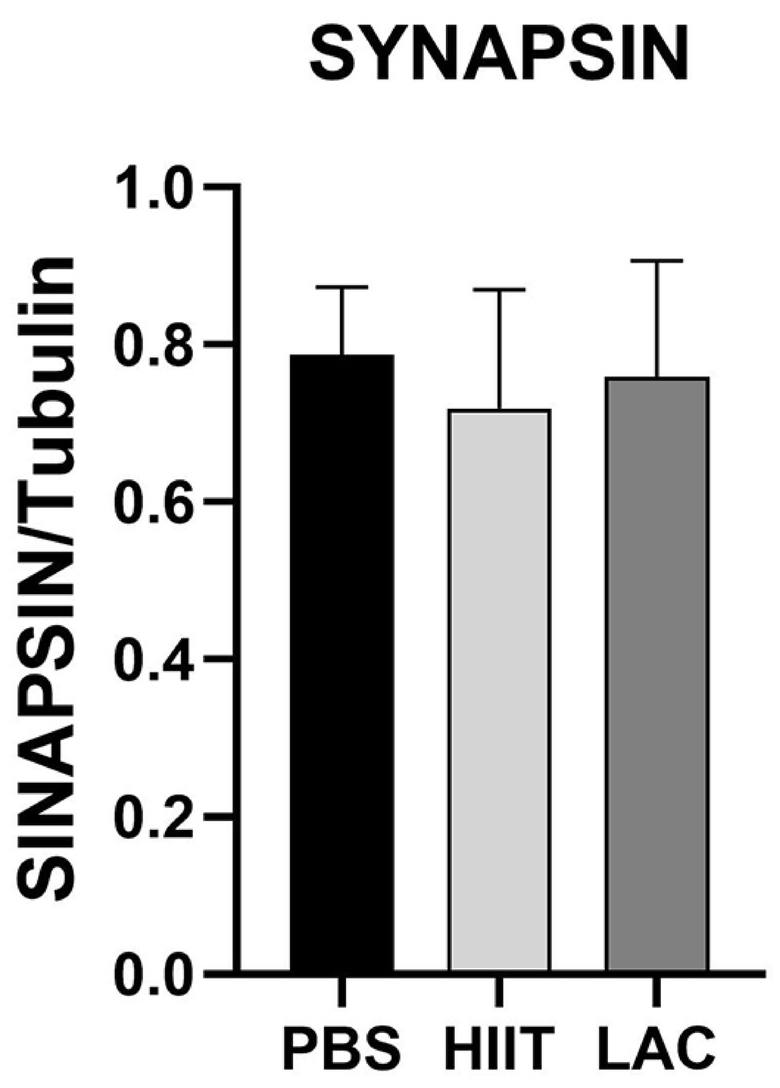
SYNAPSIN
SINAPSIN/Tubulin
1.0
0.8
0.6
0.4
0.2
0.0
PBS
HIIT
LAC

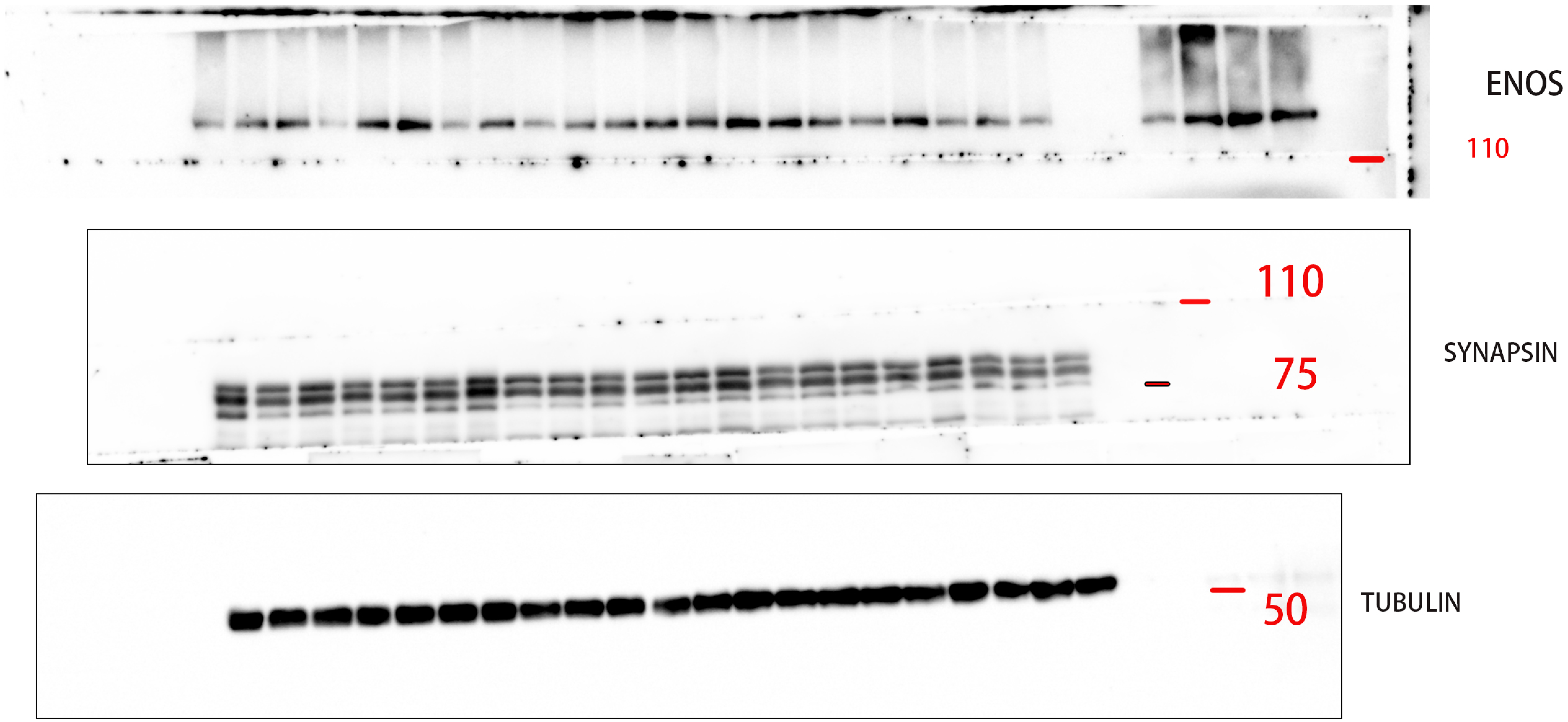
ENOS
110
110
75
SYNAPSIN
50
TUBULIN

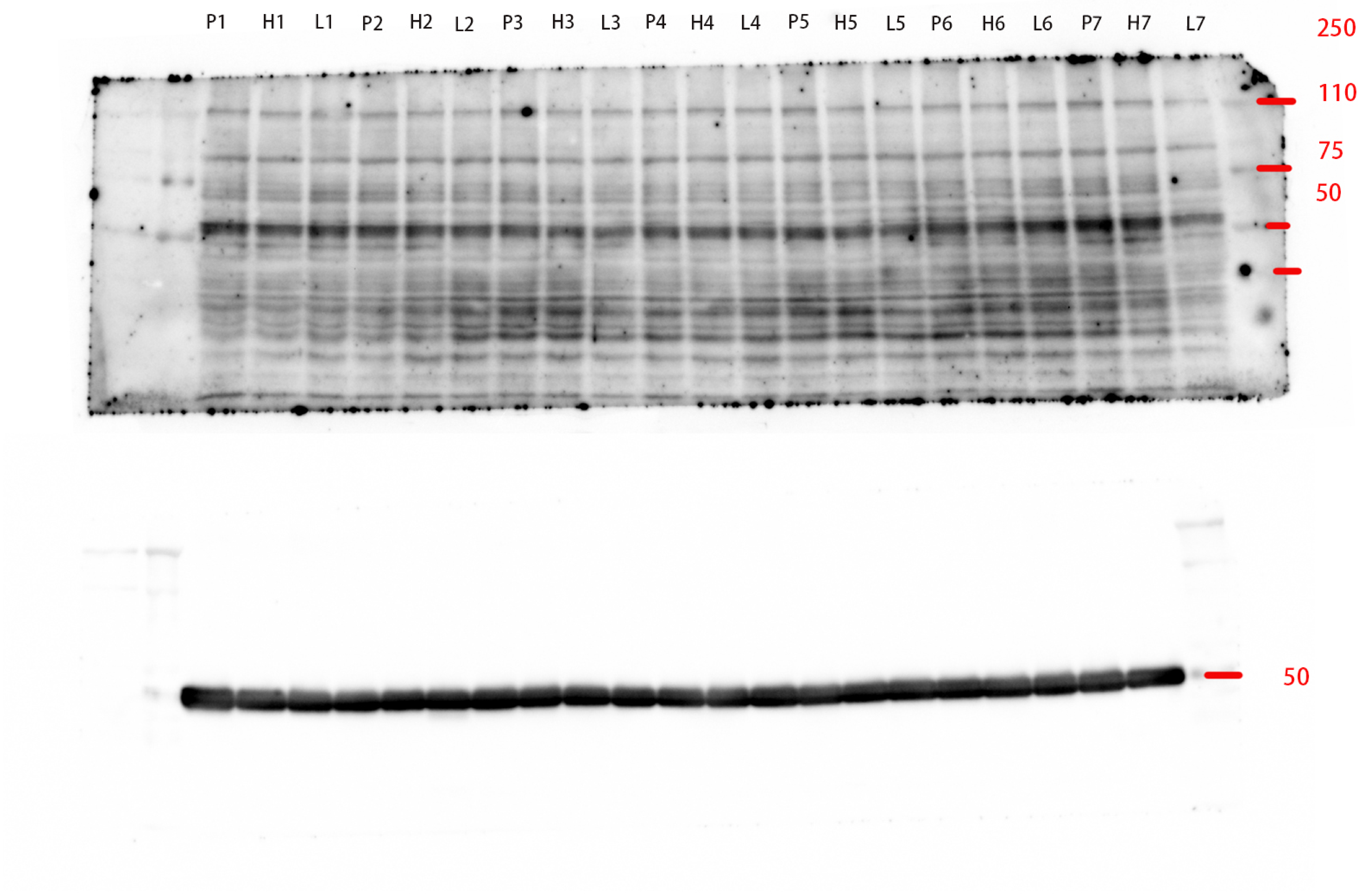

Pan Lactic acid-Lysine

TUBULIN

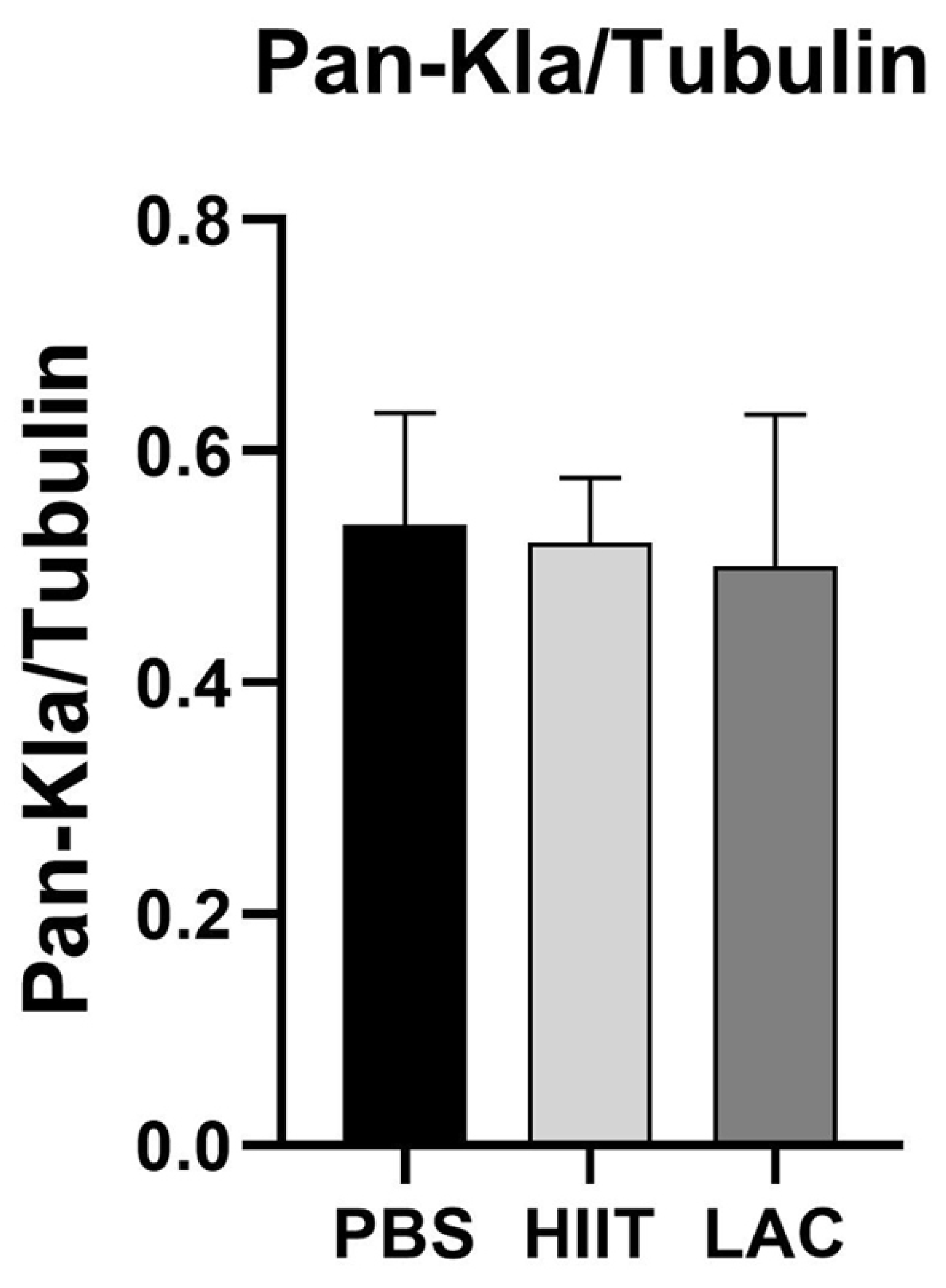

Supplement: Multimedia component 2 [file mmc2.pdf]
